# Focal High-Grade Areas with a Tumor-in-Tumor Pattern: Another Feature of Pediatric *DICER1*-Associated Thyroid Carcinoma?

**DOI:** 10.1007/s12022-025-09863-2

**Published:** 2025-05-31

**Authors:** Marco Schiavo Lena, María Sánchez-Ares, Emanuela Brunetto, Ihab Abdulkader-Nallib, Riccardo Maggiore, Diego Barbieri, Maria Cristina Vigone, Francesca Perticone, Roberto Lanzi, Silvia Presi, Paola Carrera, Maria Giulia Cangi, Gianluigi Arrigoni, Claudio Doglioni, José Manuel Cameselle-Teijeiro

**Affiliations:** 1 Pathology Unit, IRCCS Ospedale San Raffaele, ENETS Center of Excellence, Via Olgettina 60, 20132 Milan, Italy; 2 Department of Pathology, Galician Healthcare Service (SERGAS), Clinical University Hospital of Santiago de Compostela, Health Research Institute of Santiago de Compostela (IDIS), University of Santiago de Compostela (USC), Santiago de Compostela, Spain; 3 Department of Surgery, Endocrine Surgery Unit, IRCCS Ospedale San Raffaele, Milan, Italy; 4 Head and Neck Department, Otorhinolaryngology Unit, IRCCS Ospedale San Raffaele, Milan, Italy; 5 Pediatric Unit, IRCCS Ospedale San Raffaele, Milan, Italy; 6 Department of Internal Medicine, Endocrine Unit, IRCCS Ospedale San Raffaele, Milan, Italy; 7 Laboratory of Clinical Molecular Genetics, Unit of Genomics for Diagnosis of Genetic Diseases, IRCCS Ospedale San Raffaele, Milan, Italy; 8 Vita e Salute University, Milan, Italy

**Keywords:** Thyroid, *DICER1*, Pediatric thyroid cancer, Tumor-in-tumor, High-grade, Poorly differentiated thyroid carcinoma, *CHEK2*, Immunohistochemistry

## Abstract

In the thyroid gland, during childhood or adolescence, *DICER1*-driven tumors include differentiated follicular thyroid carcinoma and, more rarely, poorly differentiated carcinoma. Herein, we describe the features of *DICER1*-associated thyroid carcinoma with the presence of high-grade areas within a differentiated tumor in four patients (median age 12.5 years, range 6–15 years), three of them carrying germline pathogenic variants of *DICER1*. A new tumor-in-tumor pattern characterized by intratumoral nodules with a higher histological grade (increased mitotic activity/Ki-67 and solid/trabecular/insular and/or microfollicular architecture) was detected in these *DICER1*-associated tumors. In two patients, the high-grade component also demonstrated the presence of *CHEK2* p.(Tyr390Cys) likely pathogenic variants, suggesting a role for this gene and more generally for the *ATM*-*CHECK2*-*TP53* pathway as a mechanism of malignant progression of *DICER1*-associated thyroid carcinomas. One of these two patients presented lymph node recurrence 8 months after surgery. An immunohistochemical study was also performed to explore the possible contribution of anti-DICER1 antibodies as well as thyroglobulin, Ki-67, p53, and PRAME in characterizing these tumors. DICER1 proved to be strongly expressed in mutated tumors compared to a control cohort (*p* < 0.001), deserving further validation to define its possible diagnostic role. Finally, well-demarcated ischemic-like areas with ghost cells embedded in a thick hyaline stroma (atrophic changes) were found within four tumors, whereas bunches of ectatic macrofollicles lined by flattened epithelium (involutional changes) were only detected in the background thyroid parenchyma of patients with germline *DICER1* variants. These morphological features may alert pathologists to suspect a somatic and/or germline *DICER1* alteration.

**Supplementary Information:**

The online version contains supplementary material available at 10.1007/s12022-025-09863-2.

## Introduction

*DICER1* syndrome is an autosomic dominant disorder, with variable penetrance, associated with a series of conditions ranging from malformations or structural anomalies (ocular, renal, dental) to hamartomatous lesions, benign, teratoid, and malignant tumors involving multiple organs [1, 2].

*DICER1* encodes a highly conserved RNaseIII endoribonuclease ubiquitously expressed in the cytoplasm. It acts as a master regulator involved in miRNA maturation, cleaving pre-miRNA hairpin into mature 5p miRNA and 3p miRNAs, which then regulate gene expression on a post-transcriptional level through mRNA degradation and repression of translation. *DICER1* alteration pattern is usually characterized by a germline loss-of-function variant and a second somatic hotspot missense variant in metal ion binding residues leading to compromised activity of domain IIIb of the protein. The consequent result is not a complete abolition of DICER1 activity, rather a decrease in the 5p:3p strand ratio since the hotspot mutations do not abrogate 3p miRNA production [3, 4].

In the thyroid gland, germline *DICER1* pathogenic variants are associated with multiple benign thyroid nodules, differentiated thyroid carcinoma, and poorly differentiated thyroid carcinoma (PDTC), while thyroblastoma is an embryonal high-grade neoplasm secondary to somatic mutations [1, 5]. *DICER1* variants are highly penetrant for goiter/thyroid follicular nodular disease (FND), especially in women: Three out of four women and one out of six men with germline *DICER1* pathogenic variants will develop FND or undergo thyroidectomy before the age of 40 [6]. Somatic *DICER1* mutations have been detected in about 3% of follicular-patterned thyroid neoplasms in adults [7], but children and adolescents with follicular-patterned thyroid carcinoma and PDTC have a higher prevalence of *DICER1* alterations [8, 9].

By the age of 20, the cumulative incidence of FND or history of thyroidectomy is 32% in women and 13% in men carrying germline *DICER1* pathogenic variants and there is a 16- to- 24-fold increased risk of thyroid cancer [6]. *DICER1* alterations are responsible for the familial association of FND and ovarian Sertoli-Leydig cell tumor [10]. The latter association has been known for many years [11], as early as in 1951 Javert and Finn mentioned a thyroid adenoma, with a morphology that was intriguingly defined as “fetal” removed from a 21-year-old female who had had an “arrhenoblastoma” 4 years previously [12].

In 2020, Chernock et al. [13] described six high-grade carcinomas that meet the World Health Organization (WHO) criteria [1] for the definition of PDTC in patients ≤ 21 years of age and found hotspot somatic pathogenic variants of *DICER1* in five of them. Moreover, a further inactivating event was identified in three patients, including one germline variant, one somatic variant, and one loss of heterozygosity. In five out of six cases, the tumor had a diffuse PDTC appearance, and in the sixth case, it was a focal feature within a tumor with well-differentiated morphology. No further recurrent alterations were detected except for *ATM* gene. Chernock [13] and other researchers [9, 14] have suggested that pediatric PDTC may have different molecular mechanisms compared to the adult counterpart and they are not always associated with poor prognosis although lymph node, distant metastases, and deaths from the disease have been reported. However, it should be underscored that currently there are no criteria for the definition of differentiated high-grade thyroid carcinoma (DHGTC) or PDTC developed specifically for pediatric patients. Rather, the criteria proposed for adults [1, 15, 16] have simply been translated to pediatric patients.

In the present series, seven tumors from four patients, including three with germline *DICER1* variants and one with hotspot somatic variant, have been described. The presence of a nodules with high-grade morphological features arising within the tumor (tumor-in-tumor pattern) was identified in five cases, expanding the morphological spectrum of *DICER1*-associated thyroid lesions. A molecular analysis was carried out both in these high-grade areas and in the background tumor, to define the evolutive potential of the former. The possible use of an anti-DICER1 antibody as well as thyroglobulin, Ki-67, p53, and PRAME was investigated. Finally, special attention was paid to the non-lesional thyroid parenchyma to characterize the features that might suggest germline *DICER1* alterations to pathologists.

## Material and Methods

### Patient Selection and Histopathological Examination

Following the publication by Chernock [13], we collected four cases of pediatric patients (≤ 18 years) with thyroid tumors showing high-grade morphological features (Table 1). All patients were Italian and underwent surgery at the Endocrine Surgery Unit of IRCCS Ospedale San Raffaele, Milan, after prior evaluation by a multidisciplinary committee. All the four surgical specimens were examined entirely following the protocol of the Pathology Unit for pediatric patients. Criteria set up by the WHO latest edition [1, 5] were adopted for all patients.

**Table 1:** Main clinical-pathological features of *DICER1*-associated tumors in this series

Pt ID	Age	Gender	Thyroid localization	Tumor ID	Diagnosis	Malignancy	Tumoral architecture	Diameter (mm)	Capsule	Capsule invasion	Vascular invasion	No. of foci vascular invasion	ETE
1	12	M	Left lobe	IA	WDTC	Carcinoma	Microfollicular	65	Yes	Yes	Yes	5	NO
1	12	M	Right lobe	IB	WDTC (?); WDTT (?)	Carcinoma (?); Borderline tumor (?)	Papillary + follicular	11	No	No	No	0	NO
2	15	M	Left lobe	II	WDTC	Carcinoma	Follicular	45	Yes	Yes	No	0	NO
3	6	F	Left lobe	IIIA	WDTC	Carcinoma	Follicular	38	Yes	Yes	Yes	1	NO
3	6	F	Right lobe	IIIB	WDTT	Borderline tumor	Papillary + follicular	7	No	No	No	0	NO
4	13	F	Isthmus	IVA	WDTC	Carcinoma	Follicular	43	Yes	Yes	No	0	NO
4	13	F	Left lobe	IVB	PDTC	Carcinoma	STI	21	Yes	Yes	Yes	2	NO
Pt ID	Tumor ID	Margin status	No. of HG foci	Focus main diameter (mm)	Mitotic count (hot spots)	Ki-67 (HG area)	Necrosis	p53	PRAME (%)	TG (pattern in HG area)	DICER1 germline variant	Follow-up (months)	
1	IA	Free of tumor	2	8	21/2 mm^2^	18%	No	WT	15	Dot-like > microfollicular	Yes	36	
1	IB	Free of tumor	1	5	8/2 mm^2^	15%	No	WT	0	Dot-like > microfollicular	Yes	36	
2	II	Free of tumor	3	8	6/2 mm^2^	9%	No	WT	0	Dot-like > microfollicular	No	8	
3	IIIA	Free of tumor	1	1	2/2 mm^2^	/	No	WT	0	/	Yes	30	
3	IIIB	Free of tumor	0	0	0/2 mm^2^	/	No	WT	0	/	Yes	30	
4	IVA	Free of tumor	1	5	4/2 mm^2^	13%	No	WT	0	Microfollicular	Yes	17	
4	IVB	Free of tumor	All the tumor	21	6/2 mm^2^	/	No	Block +	20	Dot-like > microfollicular	Yes	17	

*Abbreviations*: *Pt* patient, *WDTC* well-differentiated thyroid carcinoma, *WDT* well-differentiated thyroid tumor, *PDTC* poorly differentiated thyroid carcinoma, *ETE* extrathyroidal extension, *HG* high histological grade, *WT* wild type, *TG* thyroglobulin

During the histological examination, particular attention was paid to two distinct features, which have been associate with *DICER1*-mutated tumors and *DICER1* syndrome respectively:

1. Atrophic changes as a well-demarcated ischemic-like area *within the tumor*, with vanishing or “ghost cells” embedded in a thick hyaline stroma, without evidence of inflammation, apoptosis or signs associated with fine-needle aspiration procedures. They are preferably found in the subcapsular region of the tumor [17, 18], can be multiple, as can be observed in multiple nodules, even small ones, outside the main lesion, but remain essentially an intranodular feature.
2. Involutional changes as a bunch of ectatic macrofollicles lined by a flattened epithelium *in the background thyroid parenchyma* [2, 19].

Nuclear features of the tumors were investigated as well and reported in descriptive terms.

The germline genetic test for *DICER1* was suggested after the histological examination due to the morphological features of the thyroid and the age of the patients to complete the diagnostic work-up. Written informed consent was obtained from the parents of all children before performing germline tests. The results of the tests, done for diagnostic purposes, were then collected retrospectively for the current study. Data were processed anonymously in order not to make any patient recognizable.

### Immunohistochemistry

Serial 5-µm-thick formalin-fixed and paraffin-embedded tissue sections were collected and processed in the Pathology Unit of IRCCS Ospedale San Raffaele. An automated immunostainer (BenchMark ULTRA; Ventana Medical System, Tucson, AZ) was used. Antibodies against the following antigens were deployed: DICER1 (cl. CL378, Atlas Antibodies, Stockholm, Sweden), thyroglobulin (cl. 2H11 + 6E1, Ventana), Ki-67 (cl. 30–9, Ventana), p53 (cl. DO7, Ventana), and PRAME (cl. EPR20330, Ventana) on whole section for each case.

The intensity of cytoplasmic expression of DICER1 was evaluated semi-quantitatively in tumors compared to the background thyroid parenchyma (from 0 to 4 +). Furthermore, the expression of DICER1 was scored in an unselected control group of 78 thyroid and parathyroid lesions (*DICER1* mutational status unknown). Two pathologists were involved in the evaluation (MSL and CD); they were blind to the mutational status, and inter-observer agreement between them was assessed using Cohen’s kappa coefficient.

Therefore, differences in DICER1 expression were assessed between (1) main tumor lesions and background thyroid tissue, using the Mann–Whitney *U* test; (2) *DICER1*-mutated tumors *versus* lesions of the control group.

Thyroglobulin staining was considered positive when there was cytoplasmic and/or colloid positivity. Ki-67 was reported as a percentage of stained nuclei in the areas of the highest intensity (hotspots). Nuclear expression of p53 was evaluated in a semi-quantitative way and reported as null-pattern if completely absent in the presence of an adequate internal control, block-pattern if diffusely and intensely positive and wild-type pattern in the presence of both negative and positive nuclei with variable intensity. PRAME expression was assessed semi-quantitatively as a percentage of positive nuclei.

### Genetic and Molecular Analysis

For germline molecular analysis, next-generation sequencing (NGS) was performed on genomic DNA extracted from blood-derived lymphocyte samples from the patients. A probe capturing enrichment method was performed using the commercial TruSight Hereditary Cancer kit (Illumina, San Diego, CA, USA), following the manufacturer’s protocols, and sequenced on NextSeq500 (Illumina). Sequencing data analyses were performed with different tools (Dragen Bio-IT, Illumina and eVai EnGenome Italy), and *DICER1* gene was analyzed alone or in a larger panel of genes in relation to the patient’s phenotype (*PTEN*, *RET*, and *MEN1* genes were also analyzed for three of the four patients). Average coverage was around 700 ×, including exon–intron junctions, and 100% of the target region had a coverage above 30 ×. Based on the evidence, annotated variants were classified into five classes according to the ACMG and AMP criteria [20]. Only pathogenic (class 5) and likely pathogenic (class 4) were reported.

For the analysis of somatic mutations through NGS, tissue samples were obtained by laser capture microdissection using the system Leica AS LMD (Leica Microsystems, Wetzlar, Germany) according to the manufacturer’s instructions.

Representative 4-μm-thick paraffin sections containing thyroid lesions stained with hematoxylin were deposited in specific frame slides for laser microdissection so that in each case, only “high-grade” and “low-grade” tumor tissue could be selected for subsequent DNA extraction as previously reported [21]. DNA was extracted using the QIAamp DNA FFPE Tissue kit (Qiagen) according to the manufacturer’s protocol. DNA quality (DNA Integrity Number, DIN) and quantity were measured using the Genomic DNA ScreenTape assay on the 4200 TapeStation system (Agilent, Santa Clara, CA, USA) according to the manufacturer. An experienced pathologist (JMCT) monitored the dissection procedure in real time.

For the custom panel (THYROSAN), custom capture RNA probes were designed using the SureDesign software web site hg19/GRCh37 (Agilent Technologies, Santa Clara, CA, USA) for targeted sequencing for all exons, 3′UTR, 5′UTR, promoter, and at least, 25 bp 5′ and 3′ flanking intronic sequences of 102 thyroid related genes for detection of single nucleotide variants (SNVs), insertions, and deletions (indels). The complete list of genes examined is provided in Table S1.

Briefly, 200 ng of genomic DNA (gDNA) was sheared on the Covaris M220 focused-ultrasonicator (Covaris Inc., Woburn, MA, USA) to generate DNA fragments between 150 and 200 bp. Libraries were prepared using the SureSelect XT HS Target Enrichment system using the Magnis NGS Prep system (Agilent Technologies) following the manufacturer’s protocol. Post-enriched libraries were validated and quantified using the High Sensitivity D1000 ScreenTape system on the 4200 TapeStation system (Agilent Technologies) according to the manufacturer’s protocol. Pooled libraries were subsequently sequenced using the NextSeq 550 Mid Output Kit v2.5 (150 cycles) on the Illumina NextSeq 550 system as 2 × 76-bp paired-end reads (Illumina, Inc., San Diego, CA, USA). This platform collects all the information in demultiplexed and paired FASTQ files for subsequent bioinformatic analysis. An average coverage of about 400 × (> 95% of the gene’s target nucleotides are covered at 200 ×, after removing the duplication reads) was obtained.

FASTQ files were aligned, and variant calling was assessed using SureCall NGS data analysis software version 4.2. (Agilent Technologies). For variant interpretation and reporting, the Alissa Interpret Analysis Software v.5.3.4 (Agilent, Santa Clara, CA, USA) was used. The following annotation source codes were used: CIViC—Clinical Interpretations of Variants in Cancer release 01-Jan-2023, NCBI ClinVar, COSMIC release v99, dbSNP build 151, JAX-CKB™—version 20240510 and gnomAD release 2.0.2. Variants were visually examined using the Integrative Genome Viewer (IGV) from the Broad Institute (http://www.broadinstitute.org/igv/).

## Results

### Clinicopathological and Molecular Findings

All patients underwent total thyroidectomy and presented globally seven tumors that were analyzed (Table 1). They were all clinically and biochemically euthyroid at the time of surgery. None of them had a previous history of malignancy, chemotherapy, or radiotherapy.

#### Patient 1 (Tumors IA and IB)

**Fig. 1:**
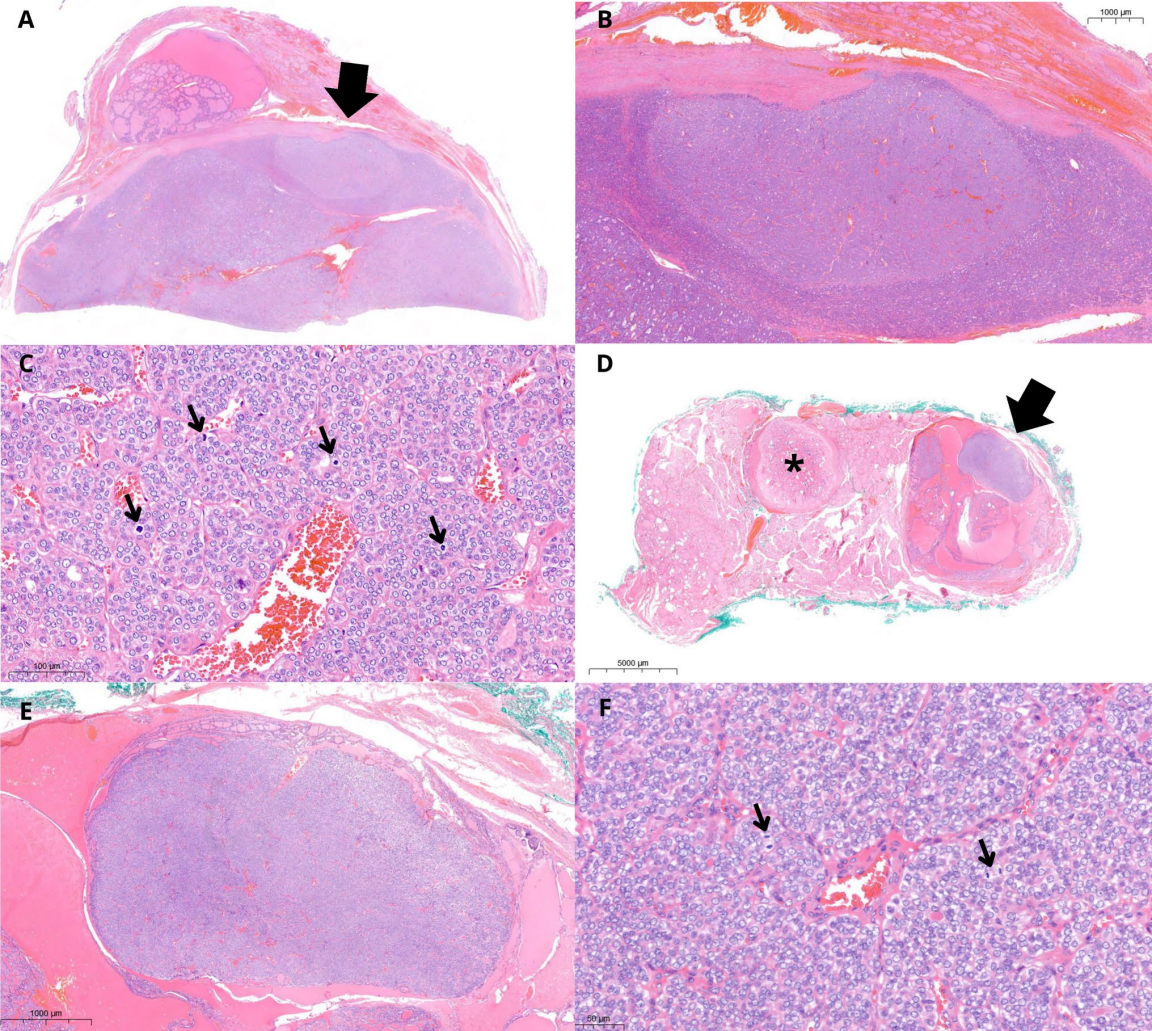
Patient 1. Hematoxylin and eosin (H&E) stain. Well-differentiated thyroid carcinoma (tumor IA) with microfollicular architecture and an 8-mm intranodular area (arrow, **A**) with solid growth and increased mitotic activity (**B**, **C**; arrows in **C** indicate mitoses). In the contralateral lobe, an 11-mm tumor (IB) inside which there was a 5-mm tumor-in-tumor pattern area (arrow, **D**) with solid growth and increased mitotic activity (**E**, **F**; arrows in **F** indicate mitoses). Atrophic changes were found in the tumor as well as in nodular-shaped areas (asterisk) in the non-lesional parenchyma of both lobes (**D**)

Patient 1 was a 12-year-old male. His 88 g thyroid gland showed a left-dominant nodule (tumor IA) measuring 65 mm and an 11-mm tumor in the right lobe (tumor IB). Tumor IA (Fig. 1) was encapsulated, had a predominantly follicular architecture, with capsular invasion and extensive venous invasion (five foci). Inside tumor IA, there were multiple areas, two of which were larger (up to 8 mm) and better defined, with a tumor-in-tumor pattern; these areas showed microfollicles evolving towards STI architecture, round and clarified nuclei, and increased mitotic activity (up to 21 mitoses/2 mm^2^) but no necrosis. Tumor IB (Fig. 1) was partly cystic and showed a papillary and follicular architecture, clarified nuclei and a mural nodule with solid architecture, and increased mitotic activity (8 mitoses/2 mm^2^) measuring 5 mm. No vascular or capsular invasion nor necrosis were detected. Atrophic changes were documented either in the 65 mm tumor (IA) and in the form of bilateral nodular areas in the non-lesional parenchyma, while they were absent in tumor IB. Involutional changes were focally detected outside the tumors, at the periphery of both lobes.

**Fig. 2:**
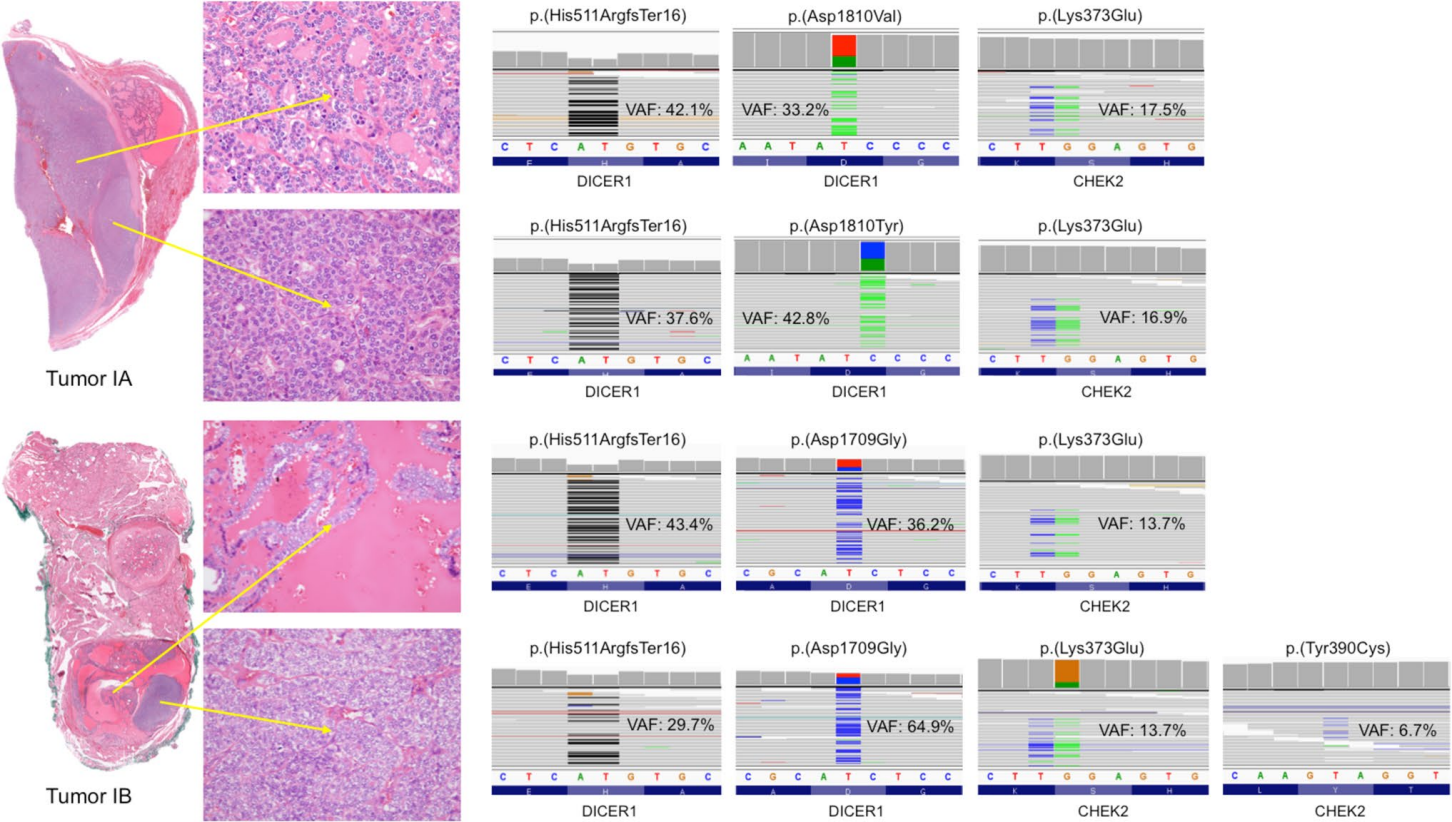
Molecular analysis of tumors IA and IB, with sub-analysis of the low- (top) and high-grade (bottom) components for both tumors

This patient showed the germline *DICER1* pathogenic variant p.(His511 ArgfsTer16), which leads to the formation of a truncated protein, as already described [22] (Table 2**)**. This variant was detected by somatic analysis in both components of tumors IA and IB (Fig. 2). Tumor IA also showed a somatic mutation of *DICER1* p.(Asp1810 Val) in the well-differentiated component, while in the 8-mm high-grade area, a *DICER1* p.(Asp1810 Tyr) missense mutation was detected. Tumor IB was characterized by the somatic pathogenic variant of *DICER1* p.(Asp1709Gly) in both components. The *CHEK2* likely pathogenic variant p.(Tyr390 Cys) was found in the high-grade nodular area of 5 mm. Furthermore, the *CHEK2* likely pathogenic variant p.(Lys373Glu) was also observed in all components of both tumors. However, no *CHEK2* germline variants were detected.

**Table 2:** Molecular findings of the high- and low-grade areas of *DICER1*-associated thyroid tumors

Pt	Tumor	Grade	*DICER1*	VAF	*CHEK2*	VAF
1	IA	Low	(G) c.1532_1533 del p.(His511 ArgfsTer16) (S) c.5429 A > T p.(Asp1810 Val)	42.1 33.2	(S) c.117 A > G p.(Lys373Glu)	17.5
		High	(G) c.1532_1533 del p.(His511 ArgfsTer16) (S) c.5428G > T p.(Asp1810 Tyr)	37.6 42.8	(S) c.117 A > G p.(Lys373Glu)	16.9
	IB	Low	(G) c.1532_1533 del p.(His511 ArgfsTer16) (S) c.5126 A > G p.(Asp1709Gly)	43.4 36.2	(S) c.117 A > G p.(Lys373Glu)	13.7
		High	(G) c.1532_1533 del p.(His511 ArgfsTer16) (S) c.5126 A > G p.(Asp1709Gly)	29.7 64.9	(S) c.117 A > G p.(Lys373Glu) (S) c.1169 A > G p.(Tyr390 Cys)	13.7 6.7
2	II	Low	(S) c.5428G > T p.(Asp1810 Tyr)	60.0	(S) c.117 A > G p.(Lys373Glu)	22.3
		High	(S) c.5428G > T p.(Asp1810 Tyr)	60.0	(S) c.117 A > G p.(Lys373Glu) (S) c.1169 A > G p.(Tyr390 Cys)	22.3 10.7
3	IIIA	Low	(G) c.745 C > T p.(Gln249 Ter) (S) c.5113G > A p.(Glu1705Lys)	52.2 39.8	(S) c.117 A > G p.(Lys373Glu) (S) c.1420 C > T p.(Arg474 Cys)	17.9 6.1
		High	(G) c.745 C > T p.(Gln249 Ter) (S) c.5113G > A p.Glu1705Lys	42.0 29.7	(S) c.117 A > G p.(Lys373Glu)	18.7
	IIIB	Low	(G) c.745 C > T p.(Gln249 Ter) (S) c.5428G > C p.(Asp1810His)	47.1 14.0	(S) c.117 A > G p.(Lys373Glu)	14.3
4	IVA	Low	(G) c.1284_1285 del p.(Lys429 AlafsTer47) (S) c.5126 A > G p.(Asp1709Gly)	41.7 41.2	WT	
		High	(G) c.1284_1285 del p.(Lys429 AlafsTer47) (S) c.5126 A > G p.(Asp1709Gly)	43.6 46.0	WT	

*Abbreviations*: *Pt* patient, *G* germline variant, *S* somatic variant, *VAF* variant allele frequency (%), *WT* wild type

After 36 months of follow-up, there was no evidence of disease recurrence.

#### Patient 2 (Tumor II)

Patient 2 was a 15-year-old male with a solid, encapsulated, 45-mm nodule (tumor II) in the left lobe, with a predominantly follicular architecture; nuclei were enlarged, clarified, and overlapping, although predominantly rounded in shape without pseudoinclusions.

Infiltration was observed throughout the tumor capsule, but no vascular invasion was detected. At least three intranodular areas were recognized within the tumor, the largest measuring up to 8 mm, with higher cell density, which were characterized by a microfollicular/solid architecture and increased mitotic activity (up to 6 mitoses/2 mm^2^). Focal subcapsular atrophic changes were present in the tumor, while they were absent in the surrounding parenchyma; involutional changes were absent as well.

**Fig. 3:**
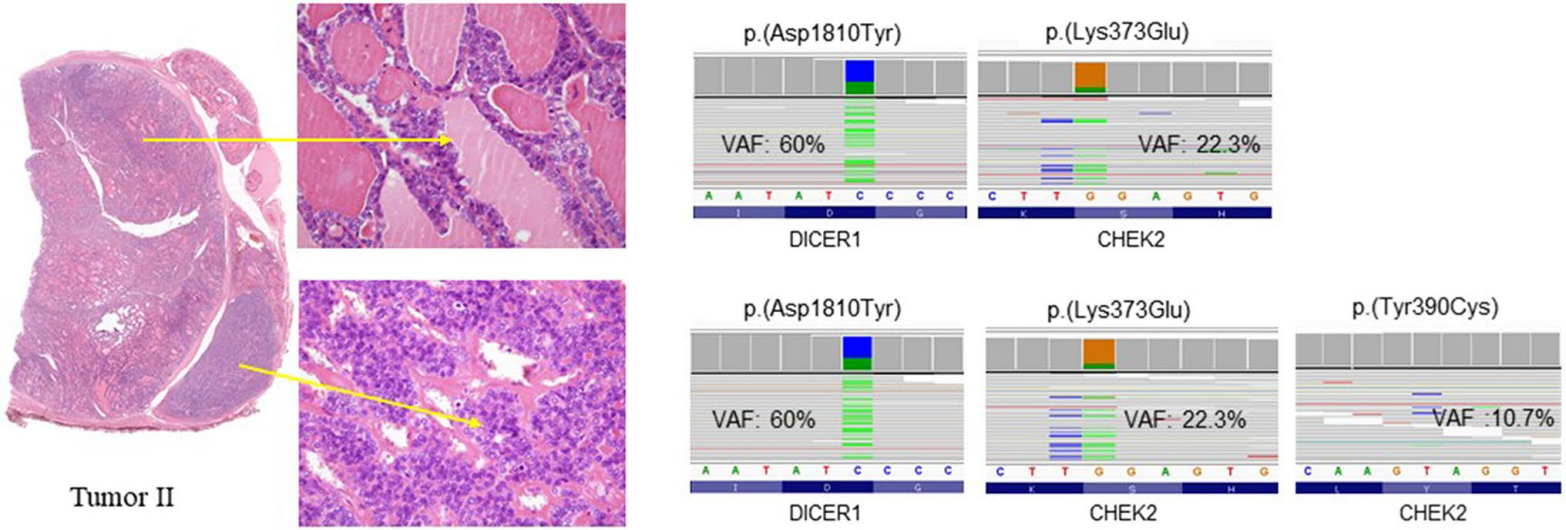
Molecular analysis of tumor II: from top to bottom, analysis of low- and high-grade component, respectively

This patient did not present germline mutations in *DICER1*, *CHEK2*, *PTEN*, *MEN1*, and *RET* genes. At a somatic level, the nodule displayed a single hotspot somatic mutation of *DICER1* p.(Asp1810 Tyr), shared by both the differentiated component of the tumor and the 8-mm high-grade area (Table 2) (Fig. 3). Additionally, the latter showed *CHEK2* p.(Tyr390 Cys) likely pathogenic variant*,* while *CHEK2* p.(Lys373Glu) likely pathogenic variant was detected in both components.

After surgery, patient 2 underwent radiotherapy with 1850 MBq of 131-I. Subsequently, 8 months after surgery, the patient had left lateral cervical lymph nodal recurrence. However, he underwent second surgery in another hospital and was lost to follow-up.

#### Patient 3 (Tumors IIIA and IIIB)

Patient 3 was a 6-year-old girl who presented, in the left thyroid lobe, a 38-mm encapsulated nodule (tumor IIIA), with either capsular and venous invasion (1 vessel). The tumor showed follicular architecture with occasional short unbranched papillae, lined by rounded nuclei, clarified with occasional grooves, as well as up to 2 mitoses/2 mm^2^. Within the tumor, a single 1-mm area of insular pattern without necrosis was identified. This area was too small to perform a reliable mitotic count. In the right lobe, there was a further 7-mm nodule (tumor IIIB), partly cystic, with a mixed papillary and follicular architecture, displaying clarified, overlapping and grooved nuclear features but with a round shape. Tumor IIIB was well demarcated but lacked a true capsule and had an entirely well-differentiated morphology. No vascular invasion nor necrosis were observed. Atrophic changes were detected either in the main tumor (IIIA) and in the surrounding parenchyma. Focal involutional changes were observed in the latter as well.

**Fig. 4:**
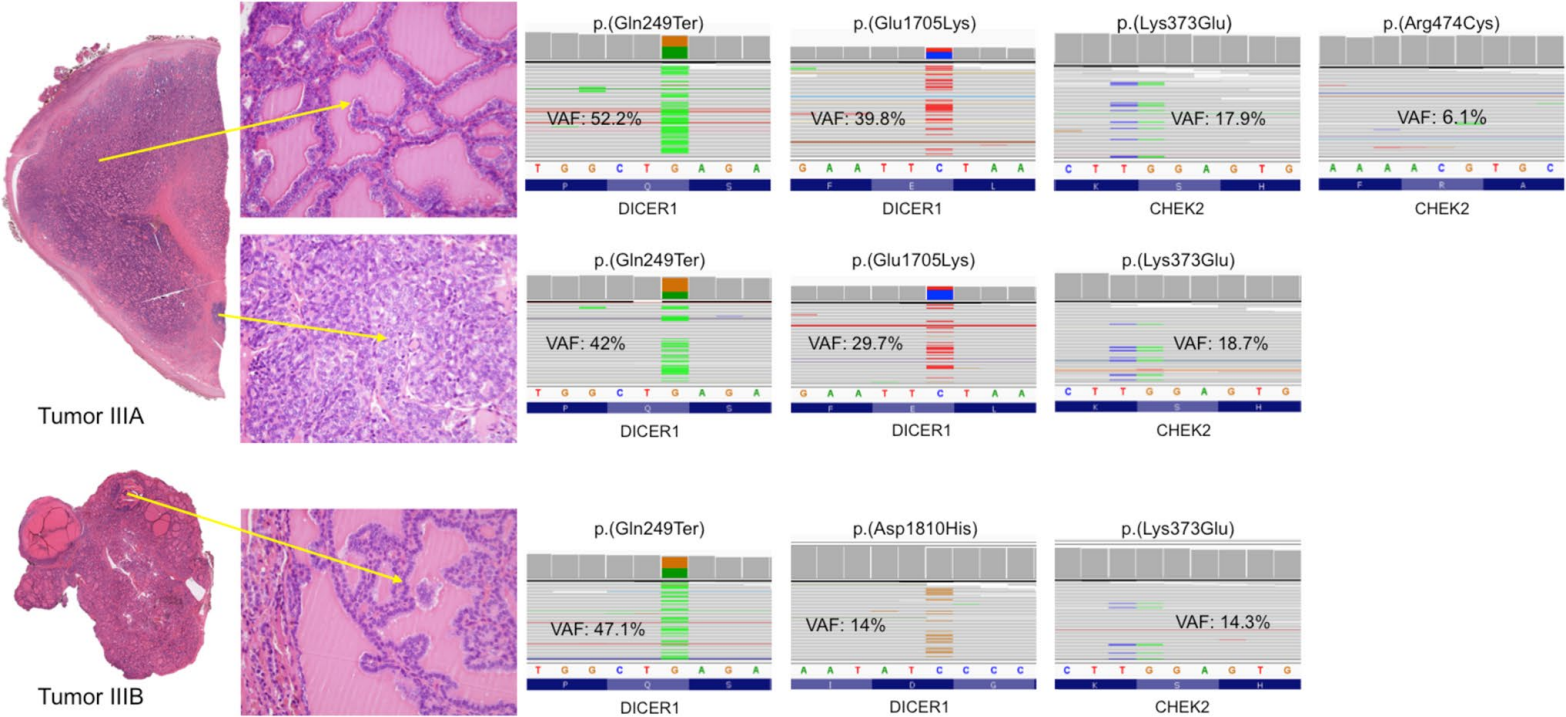
Molecular analysis of tumors IIIA and IIIIB, with sub-analysis of the low- (top) and high-grade (bottom) components for both tumors

In patient 3, the pathogenic germline variant of *DICER1* p.(Gln249 Ter) was documented. In tumor IIIA, a somatic *DICER1* pathogenic variant p.(Glu1705Lys) was also found in both components, along with a *CHEK2* p.(Lys373Glu) likely pathogenic variant. *CHEK2* p.(Arg474 Cys) likely pathogenic variant was observed only in the well-differentiated component of the tumor. Tumor IIIB showed *DICER1* p.(Asp1810His) pathogenic variant and *CHEK2* p.(Lys373Glu) likely pathogenic variant (Table 2) (Fig. 4). No *CHEK2* germline variants were observed.

Thirty months of follow-up were uneventful.

#### Patient 4 (Tumors IVA and IVB)

**Fig. 5:**
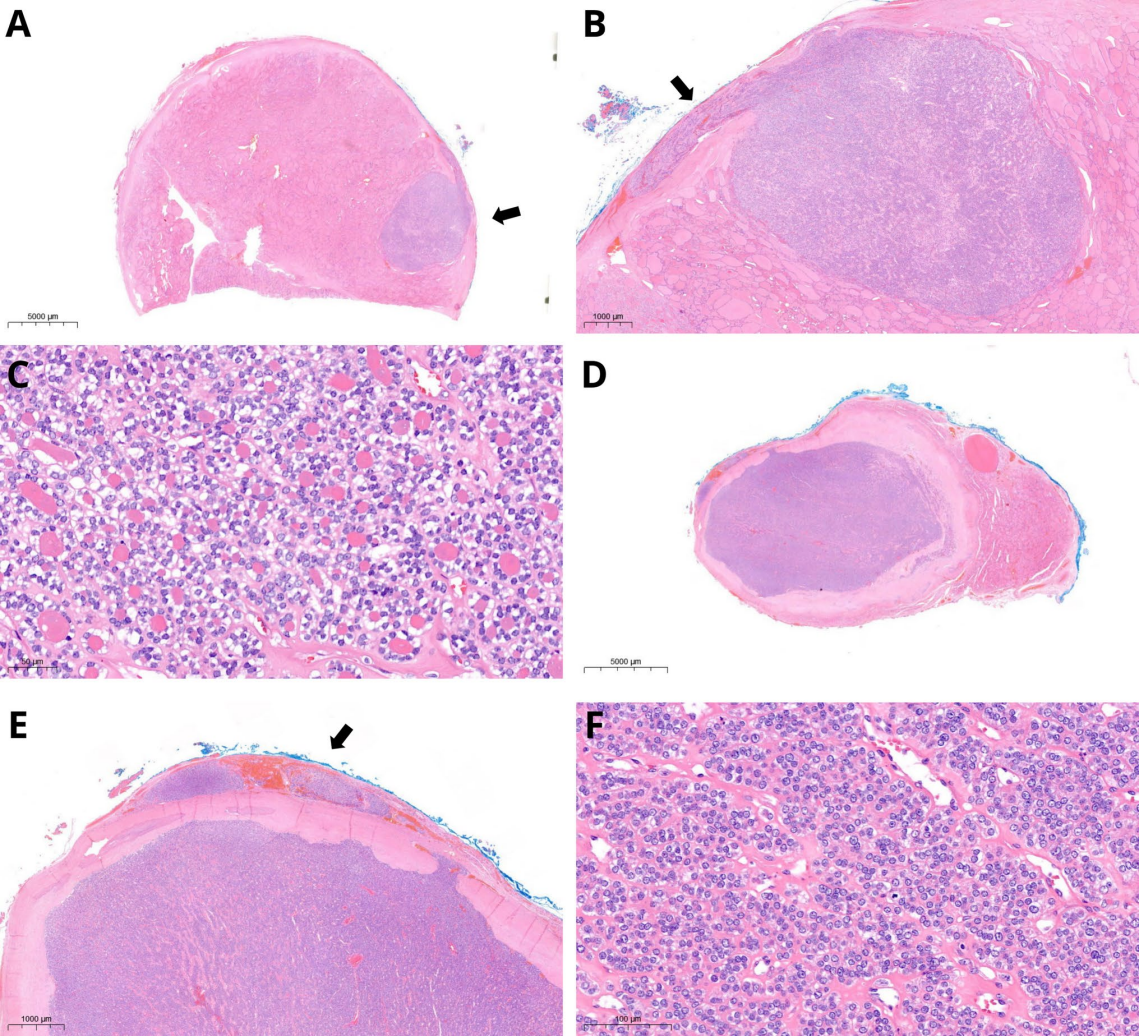
Patient 4. H&E stain. Tumor IVA. A 43-mm paraisthmic tumor showed a 5-mm intranodular area (arrow, **A**), from which capsular invasion started (arrow, **B**). Higher cellularity and microfollicular architecture (**C**) characterized the intranodular area. Tumor IVB was surrounded by a thick calcific capsule (**D**), with evident capsular and vascular invasion (arrow, **E**) and solid/trabecular/insular architecture (**F**)

The fourth patient was a 13-year-old female. Her 49 g thyroid showed in the right paraisthmic region a solid, encapsulated tumor (IVA), measuring 43 mm with infiltration of the capsule but without evidence of vascular invasion. Within the tumor, an abrupt nodular area showing a significantly higher cell density and a microfollicular architecture of 5 mm and up to 4 mitoses/2 mm^2^ was recognizable. This area extended towards the capsule, infiltrating it thoroughly. Discrete chromatin clearing and some nuclear overlapping were detected in both components of tumor IVA (Fig. 5). In the left lobe, there was a solid nodule (tumor IVB) of 21 mm with a thick, calcified capsule, showing entirely STI growth pattern, along with capsular and vascular invasion (2 vessels); the mitotic count was up to 6 mitoses/2 mm^2^. No necrosis was detected.

Within the tumor IVA, frequent atrophic changes were observed in the subcapsular area, as well as nodules in the adjacent parenchyma, while they were not present in the tumor IVB. Involutional changes were only focally detected at the polar extremities of both lobes.

**Fig. 6:**
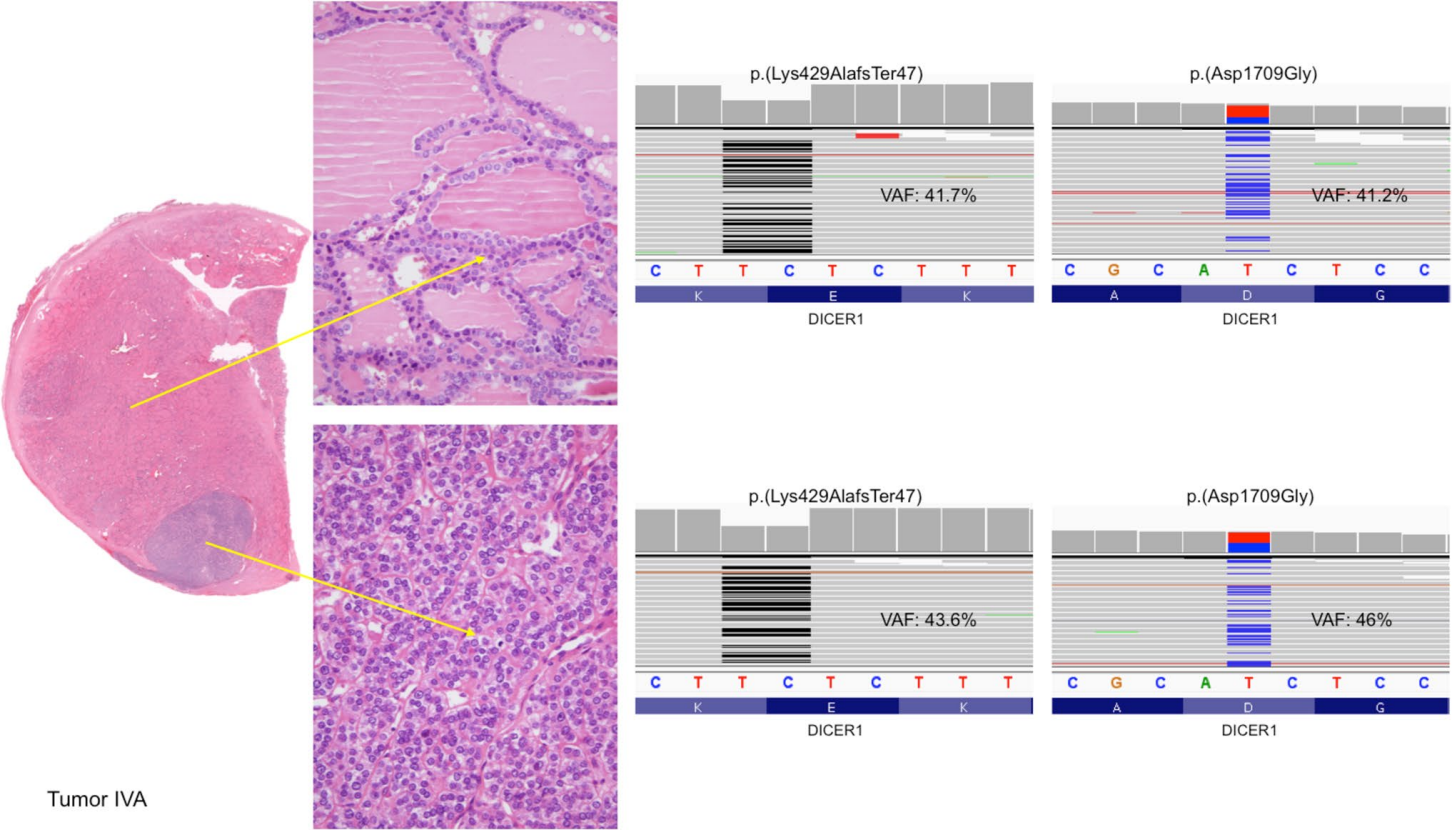
Molecular analysis of tumor IVA: from top to bottom, analysis of low- and high-grade component, respectively

Patient 4 had a germline *DICER1* p.(Lys429 AlafsTer47) pathogenic variant, which encodes a truncated protein (Table 2) (Fig. 6). Tumor IVA also revealed somatic *DICER1* p.(Asp1709Gly) pathogenic variant in both tumor components, with no further molecular alterations. For tumor IVB, genetic analysis proved to be unsuccessful due to the thick calcification of the capsule, which had required previous immersion in an acid decalcifying solution.

Patient 4 had the most limited follow-up, with no events reported 17 months after surgery.

### Immunohistochemical Findings

#### DICER1

**Fig. 7:**
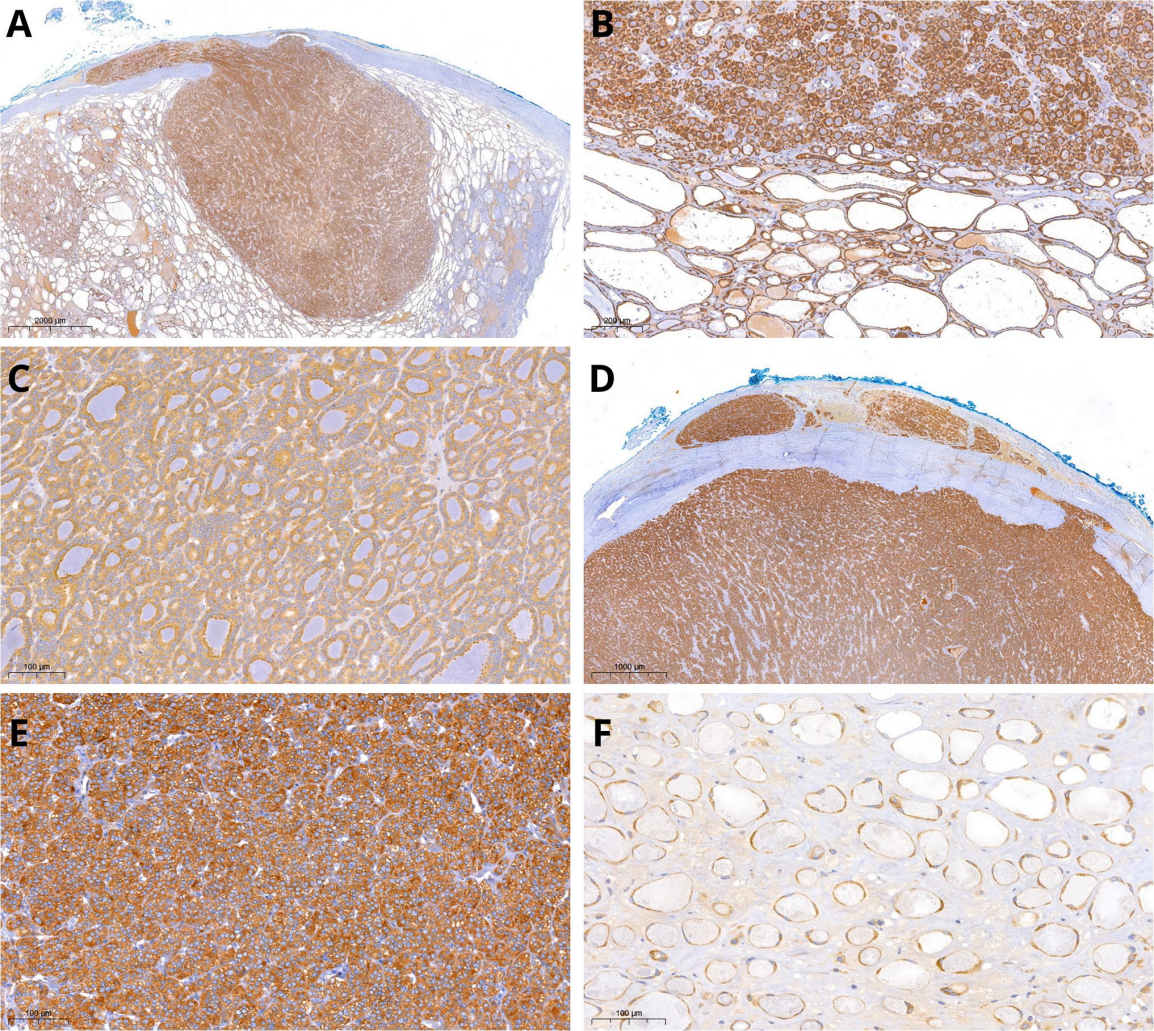
DICER1-immunohistochemistry in DICER1-associated tumors. DICER1 expression was widespread and intense with stronger stain in the high-grade component (**A**, **B**) compared to the low-grade component (**C**). Tumor IVB showed uniform, intense (4+) and diffuse positivity (**D**, **E**). Faint positivity was observed in follicles within the atrophic changes (**F**)

All seven tumors showed widespread, intense reactivity (3 + to 4 +), with stronger DICER1 expression in carcinomas and larger tumors than in the surrounding parenchyma (Table 3) (Fig. 7). The intensity of DICER1 expression was found to be significantly higher in patients with *DICER1* alterations (Mann–Whitney test, *p* < 0.001) compared to the control group with a substantial agreement in scoring between the two pathologists involved (Cohen’s *k* = 0.71).

**Table 3:** Immunohistochemical expression of DICER1

Age	Gender	Pathology	DICER1 stain intensity (main nodule/lesion)	DICER1 stain intensity (background thyroid parenchyma)
12	M	Patient 1, tumor IA	4	1
12	M	Patient 1, tumor IB	3	1
15	M	Patient 2, tumor II	3	1
6	F	Patient 3, tumor IIIA	3	1
6	F	Patient 3, tumor IIIB	2	1
13	F	Patient 4, tumor IVA	3	1
13	F	Patient 4, tumor IVB	3	1
31	F	PTC	1	1
80	F	OC	1	0
13	F	PTC	0	1
61	M	OC	1	1
42	F	NIFTP	2	1
16	M	FA	1	1
65	M	FND	1	1
49	F	HT	1	1
18	M	HT	1	1
57	M	FA	1	1
1	M	MTC	3	1
60	F	FND in PTEN/HTS	2	1
59	F	MTC	3	1
77	F	OC	3	2
70	M	MTC	3	1
40	F	DH	1	1
33	F	WDT-UMP	2	1
48	F	NFTP	1	1
86	M	MTC	3	1
56	F	PTC and DH	1	1
73	F	FTC	0	0
74	M	FND	1	1
67	F	AC	3	Absent
59	F	PA	0	Absent
58	F	SCN	2	1
35	F	DH	1	1
28	F	OC	1	1
71	M	PDTC	2	Absent
23	F	FTC	4	1
56	F	FND	1	1
61	F	MTC	2	1
41	F	FA	1	1
83	F	AC	2	1
41	F	FND	1	1
75	F	OC	1	1
51	F	FTA	1	1
57	F	FND	1	1
63	M	FA	1	1
50	F	DH	1	1
51	F	FA	3	1
64	F	PTC	1	1
60	F	FTC	0	0
50	F	FA	2	1
63	F	PTC	2	1
56	F	PTC	0	0
75	F	PTC	1	1
49	F	PTC	1	1
31	M	PTC	2	1
14	F	DH	1	1
11	M	FND	3	1
42	F	FA	3	1
24	M	PTC	2	1
68	F	PA	1	Absent
43	F	FA	0	0
62	F	PTC	2	1
43	M	MTC	3	1
51	F	FND	2	1
53	F	FA	1	1
63	F	HT	1	1
48	M	OA	2	1
71	F	PTC	1	1
86	F	PA	2	Absent
68	F	PA	1	Absent
40	M	MTC	3	Absent
65	F	DH	2	1
71	F	PA	1	Absent
74	F	PA	1	Absent
61	F	PTC	2	1
47	M	PTC	1	1
51	F	MTC	3	1
19	F	PTC	1	1
19	F	KC	1	Absent
36	F	DH	1	1
38	M	PTC	1	1
63	F	OC	1	1
78	M	PA	1	Absent
47	F	OA	1	1
59	M	PA	2	Absent

*Abbreviations*: *F* female, *M* male, *PTC* papillary thyroid carcinoma, *OC* oncocytic carcinoma, *NIFTP* non-invasive follicular thyroid neoplasm with papillary-like nuclear features, *FA* follicular adenoma, *FND* follicular nodular disease, *HT* Hashimoto thyroiditis, *MTC* medullary thyroid carcinoma, *PTEN/HTS* PTEN/hamartoma tumor syndrome, *DH* diffuse hyperplasia, *WDT-UMP* well-differentiated tumor of uncertain malignant potential, *FTC* follicular thyroid carcinoma, *AC* anaplastic carcinoma, *PA* parathyroid adenoma, *SCN* solid cell nest, *PDTC* poorly differentiated thyroid carcinoma, *OA* oncocytic adenoma, *KC* Kürsteiner cyst

The results of the control group highlighted how DICER1 tended to be expressed in various types of thyroid lesions, with an intensity equal to or slightly greater where a nodular lesion was present compared to the surrounding parenchyma (Mann–Whitney test, *p* < 0.001). Medullary thyroid carcinoma showed intense (3 +) reactivity. DICER1 positivity was also very intense (3 +) in the germinal centers of lymphocytic thyroiditis (Table 3) (Figure S1).

#### Thyroglobulin

As expected, thyroglobulin was expressed in all tumors evaluated. In tumors IA, IB, II, and IVB, the expression pattern in the high-grade areas was dot-like perinuclear with a variable presence of microfollicles (Figure S2); the microfollicular pattern was particularly rare in tumor IVB, in sharp contrast with a widespread cytoplasmatic pattern of the background thyroid. In tumor IIIA, the high-grade focus was very limited, so it was not possible to evaluate the stain; tumor IIIB showed the usual pattern of reactivity. In tumor IVA, the staining highlighted the predominant microfollicular architecture.

#### Ki-67 Proliferative Index

Ki-67 index was evaluated in the high-grade areas of tumors IA, IB, II, and IVA and showed a mean value of 13.8% (range 9–18%, median 14%). Tumor IIIA did not have sufficient material for evaluation, tumor IIIB did not have high-grade components, and tumor IVB did not provide reliable results, even after repeating the staining on several blocks, probably due to the decalcifying procedure.

#### p53

Immunoreactivity for p53 was evaluable in all cases. Only tumor IVB showed strong, diffuse positivity in > 80% of cells. In all other tumors, the reactivity pattern was wild-type, with a tendency towards greater reactivity (both more widespread and more intense) within the nodular areas of high-grade morphology (Figure S2).

#### PRAME

PRAME immunohistochemical expression was detected in two of the seven tumors studied. In tumor IA, focal (heterogeneous) nuclear positivity was observed in up to 15% of cells, both in the well-differentiated component and in the 8-mm high-grade area. In tumor IVB, with PDTC-type morphology, intense, heterogeneous, nuclear reactivity was also detected in up to 20% of cells, which was most intense in the subcapsular region (Figure S2). All other tumors were completely negative.

## Discussion

Since the first association between pleuro-pulmonary blastoma and *DICER1* alterations in 2009 [23], many tumor histotypes have been characterized by somatic and/or germline *DICER1* pathogenic variants. Despite the great variability of tumors associated with *DICER1*, a series of recurrent morphological patterns have also been identified. In particular, several authors [17, 24] have documented the presence of somatic *DICER1* variants in encapsulated thyroid tumors with macrofollicular architecture and lack of vascular invasion, typically occurring in young women, with an excellent prognosis. At the other end of the spectrum, *DICER1*-associated thyroid tumors have been reported in pediatric/young adults showing histopathological features consistent with PDTC, including STI growth pattern, increased mitotic activity, necrosis, extensive vascular invasion, and lymph node/distant metastases [13, 14, 25].

In this study, we analyzed the characteristics of seven *DICER1*-associated thyroid tumors detected in four pediatric patients, three of them with germline pathogenic variants; the tumors were well-circumscribed or encapsulated, but capsular and/or vascular invasion was detected in five (Table 1). Within five tumors, we found microscopic nodules of 1 to 8 mm in diameter with increased cellularity and high histological grade, characterized by a STI growth pattern and increased mitotic activity that ranged from 4 to 21 mitoses per 2 mm^2^ (tumor-in-tumor pattern). Despite the absence of necrosis, these high-grade foci met the minimal criteria for PDTC in adults [1, 15].

Similar cases have already been illustrated in literature though: An insular area within a papillary carcinoma of a child was reported by Albores-Saavedra et al. [26]. A minimally invasive follicular thyroid carcinoma with a solid and microglandular growth pattern along with numerous mitotic figures was described in an 18-year-old female with a germline *DICER1* variant [27]. More recently, high-grade thyroid tumors in children and young adults have been associated with germline and somatic *DICER1* variants [9, 13, 14, 25]. The coexistence of these high-grade foci within differentiated carcinomas of our series suggests the possibility of clonal progression to PDTC.

Studies on FND associated with *DICER1* mutations have highlighted how the individual hyperplastic/adenomatous nodules showed different hotspot somatic variants in addition to the same germline variant [28]. These observations demonstrated both the clonal nature of these benign nodules and that the second somatic hit of *DICER1* is an early clonal event. Tumor IA of our series, however, showed two different somatic pathogenic variants of *DICER1* in the high- and low-grade component (Table 2), suggesting the possibility of two independent clonal events.

In the present study, the high-grade areas, after being carefully isolated, were investigated with a large gene panel to identify possible mutational mechanisms of progression and recurrent *CHEK2* likely pathogenic variants were documented.

*CHEK2* (checkpoint kinase 2) encodes for a checkpoint serine-threonine kinase protein that is activated after DNA double-strand breaks and prevents cells from entering mitosis. It is involved in cell cycle regulation, apoptosis, and is an important link between ATM/ATR kinases and downstream effectors during DNA-damage response [29]. CHEK2 serves as a phosphorylation target of ATM after DNA double-strand breaks and subsequently activates BRCA1 and p53 and inactivates CDC25 [29]. *CHEK2* germline pathogenic variants have generally been associated with a moderate increased risk of breast cancer during life in *BRCA1/2* negative patients [30], although an increased risk for syndromic familial non-medullary thyroid carcinomas has also been suggested [31]. Studies from Poland have shown that premature protein-truncating alleles (c.1100 del, splice site variant c.444 + 1G > A, also known as IVS2 + 1G > A and del5395) are associated with a greater risk of thyroid carcinoma compared to missense variant c.470 T > C,p.(Ile157 Thr) [32, 33]. Publications from other geographic areas have confirmed only a modest risk increase in non-medullary thyroid carcinomas for monoallelic *CHEK2* pathogenic variants [34, 35].

Interestingly, when we evaluated the tumor-in-tumor pattern to investigate clonal evolution, we found that the *CHEK2* p.(Tyr390 Cys) likely pathogenic variant was present in the high-grade areas of two tumors (IB and II), one of which subsequently developed a lymph node metastasis (Table 2).

The codon change from tyrosine to cysteine at residue 390 (c.1169 A > G, p.(Tyr390 Cys)), a highly conserved residue located within *CHEK2* activation loop (residues 371–390), which is part of the polypeptide substrate-binding site and contains the activating phosphorylation site, appeared to significantly impair *CHEK2* activity [36]. Functional analysis suggested that p.(Tyr390 Cys) variant is deleterious as evidenced by the mutant protein inability to inactivate CDC25 A or to activate p53 after DNA damage [37]. Cells expressing the p.(Tyr390 Cys) variant showed impaired p21 and Puma expression after DNA damage, meaning that the deregulated cell cycle checkpoint and apoptotic response may help conserve mutations and therefore contribute to tumorigenesis. In studies from China [37] and Turkey [38], *CHEK2* p.(Tyr390 Cys) missense variant was associated with breast cancer and was predicted to be most likely deleterious by Align-GVGD and SIFT and probably damaging by Poly-Phen2 [38].

In the current study, two other *CHEK2* variants were identified. The *CHEK2* c.117 A > G, p.(Lys373Glu) likely pathogenic variant was detected in multiple tumors (IA, IB, II, IIIA, and IIIB) in three patients, and it was present in both low- and high-grade components with an unclear clinical meaning. Although data are limited, it has been proposed that this variant significantly impairs the activity of *CHEK2* [39], which could have favored the development of the tumors in our series.

Conversely, since the *CHEK2* p.(Arg474 Cys) variant was present in the low-grade but absent in the high-grade component of tumor IIIA in patient 3, it seems unlikely that it is involved in disease progression.

It is relevant that a not further specified *CHEK2* variant was found, coupled with a *TP53* alteration, in a PDTC with pathogenic variant of *DICER1* in a 70-year-old man [40]. In Chernock’s series [13], the only other alteration present in more than one case besides *DICER1* was inactivation of *ATM*. Furthermore, their case no. 4 presented a pathogenic variant of *TP53*. In Whaley’s study, two *DICER1*-mutated PDTCs also displayed *TP53* mutations [14], as did two cases in Bhele’s series [40].

These data, albeit in a limited number of cases, suggested that the impairment of *ATM-CHEK2-TP53* pathway could be a mechanism involved in the malignant progression of *DICER1*-associated thyroid tumors. These findings also confirm the different molecular profile of pediatric PDTC compared to adult-onset PDTC, although *DICER1* and *ATM-CHEK2-TP53* pathway could be implicated in at least a subset of adult-onset PDTC [1; 40].

However, the mechanisms of tumor progression in *DICER1*-associated tumors may be driven by gene expression variations without alteration in the DNA sequence. The directional shift from 5 to 3p miRNA expression seems to be a necessary step in *DICER1*-associated tumorigenesis [41]. This dysregulation has already been identified in follicular adenomas, despite the non-infiltrative nature of these tumors [42]. Interestingly, a study that profiled the miRNA and mRNA transcriptomes of *DICER1*-associated thyroid tumors observed that malignant tumors (carcinomas) had an increase in 3p miRNAs, whereas this did not occur in adenomas and this fact could be at least in part explained by an increased expression of DICER1 mRNA and protein levels as observed in carcinomas [43].

In addition to the molecular landscape, the morphology of *DICER1*-driven tumors seems to be peculiar as well. Tumor nuclei were found to be larger than those of conventional follicular tumors, with chromatin clearing, slightly irregular contour, some crowding but without deep invaginations of the nuclear membrane or pseudoinclusions. These “intermediate” nuclear features, following the terminology used by Sobrinho-Simões’ group [44], were identified in all of the well-differentiated tumors in our series, highlighting the difficulties in classifying these non-*BRAF* and non-*RAS* tumors [45]. Other authors have also acknowledged difficulties in the differential diagnosis between follicular and papillary thyroid carcinoma [19, 46, 47]. Therefore, we endorsed that the designation of *DICER1*-associated well-differentiated thyroid carcinoma/tumor, in accordance with the presence/absence of invasive features respectively, is a more appropriate terminology for these *DICER1*-associated lesions [44].

In this regard, the true nature of tumor IB remains elusive, because although it was well circumscribed and without vascular invasion, it had a high-grade area harboring the *CHEK2* p.(Tyr390 Cys) likely pathogenic variant.

A recent letter on *Thyroid* has highlighted the critical need to identify those morphological features that may be indicative of *DICER1* alterations [48].

**Fig. 8:**
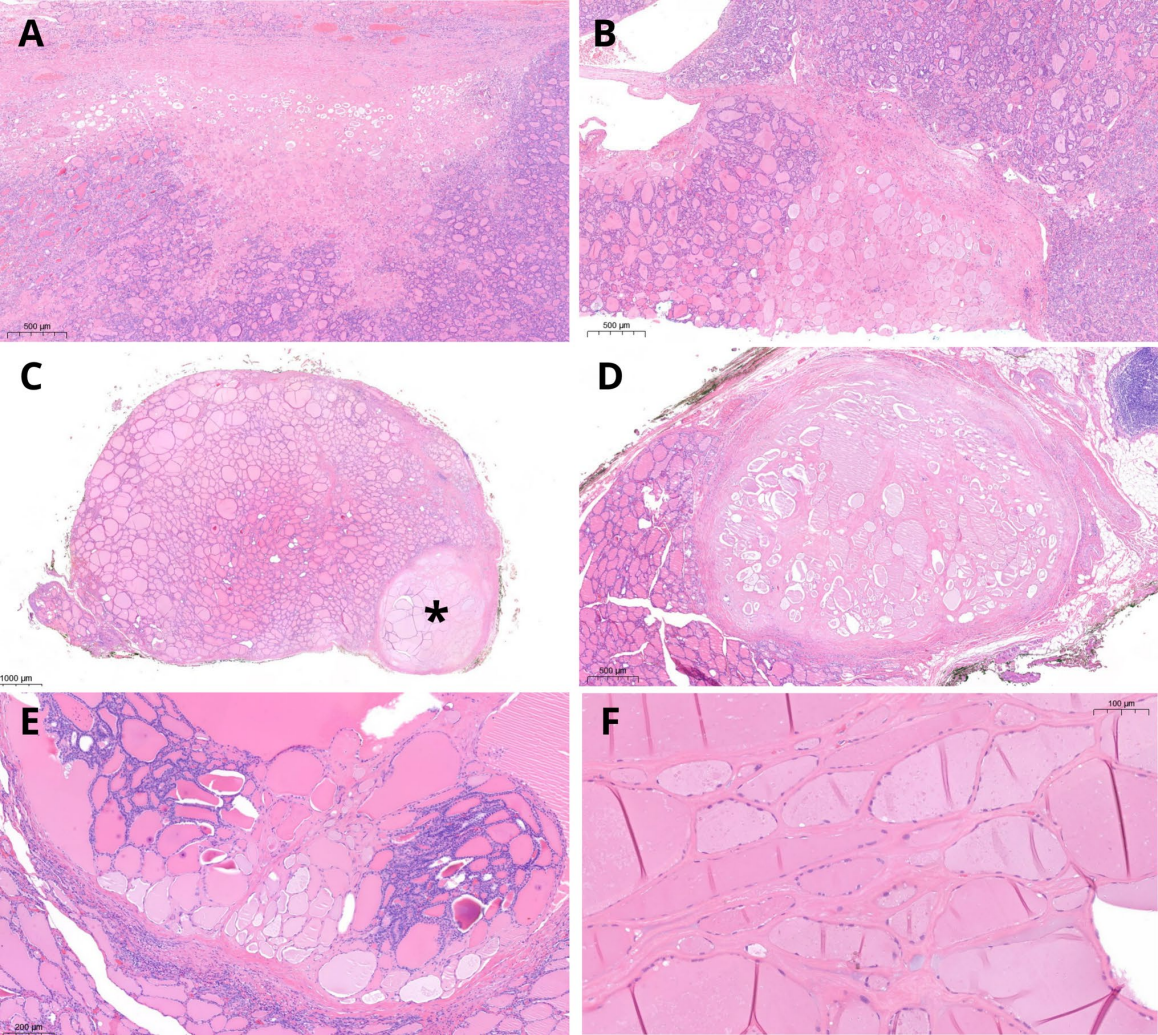
Atrophic changes. H&E stain. They are essentially an intranodular feature and represent ischemic-like, intensely eosinophilic areas, usually localized in the subcapsular zone (**A**) or more deeply within the tumor (**B**). In patients with *DICER1* germline pathogenic variant, atrophic changes can also be observed as additional nodules besides the main tumor (**C**, **D**); sometimes, a non-atrophic component is still recognizable (**E**). At higher magnification, atrophic changes are characterized by “ghost follicles” lined by flattened thyrocytes immersed in a hyaline stroma (**F**)

Considering isolated nodular lesions, some features have been associated with *DICER1* alterations, either germline or somatic. These tumors have a generic *RAS*-like phenotype, showing encapsulation, with or without invasion. The architecture is heterogeneous and includes a macrofollicular, microfollicular or mixed growth pattern. Papillary infoldings are common, but have low specificity [18]. Atrophic changes, which can be multiple in the same nodule and are typically arranged in a subcapsular location, have been shown to be a sensitive and specific marker (77% and 95%, respectively) of *DICER1* alterations [18]. However, a subsequent Asian study found much lower specificity [49]. In the present study, atrophic changes were documented in four of seven tumors (IA, II, IIIA, and IVA), with multiple changes occurring within the same tumor (Fig. 8). Tumor IVB did not display atrophic changes, since they appear to be underrepresented in PDTC [14].

**Fig. 9:**
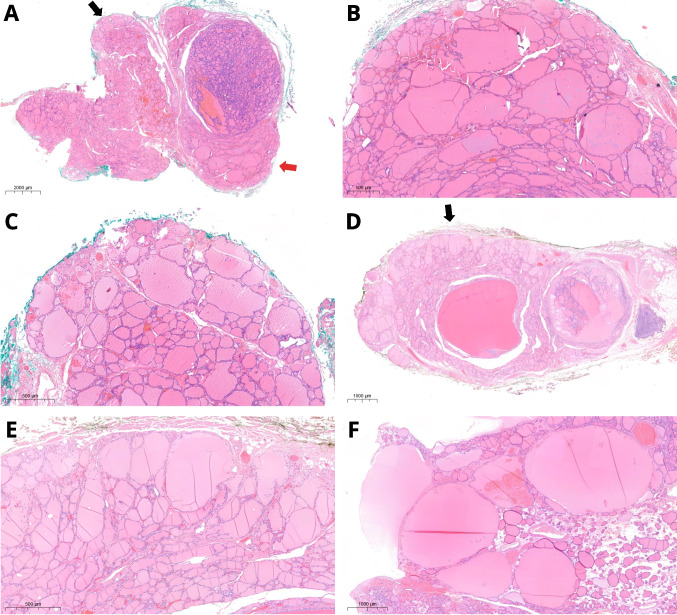
Involutional changes. H&E stain. Thyroid outside the main lesions of patient 1 showed a hyperplastic nodule and peripheral subcapsular rim with slightly ectatic follicles (arrows, **A**). Higher magnification of the areas indicated by the black (**B**) and red (**C**) arrows shows these peripheral dilated macrofollicles, lined by flattened thyrocytes. Similar findings were also observed in patient 3, with an *emphysematous-like* pattern (**D)**. A higher magnification of the spot indicated by the black arrow (**E**) and another section from the same patient (**F**)

**Fig. 10:**
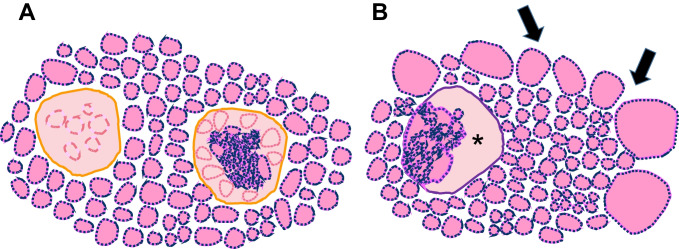
Schematic representation of the background thyroid of patient with germline *DICER1* pathogenic variants. Atrophic changes (**A**) assume a nodular configuration; a residual hyperplastic component is depicted in the nodule on the right, while a more marked atrophy affects the nodule on the left. Involutional changes (**B**) are ectatic follicles lined by flattened epithelium; if present at the periphery (arrows), they impart an “emphysematous-like” configuration which slightly deforms the outline of the section. A nodule with papillary centripetal growth and cystic component (asterisk) is also shown in the same plane of section

In the thyroid parenchyma outside the main nodule, there are some findings that may suggest *DICER1* syndrome, such as the presence of multiple smaller nodules with atrophic changes, centripetal papillary growth nodules, and involutional changes. The latter are aggregates of cystically-dilated macrofollicles, which in our cases seemed to be concentrated at the periphery of tissue section imparting an “emphysematous-like” appearance. This pattern of follicular dilatation was present in all three patients with germline mutations (Fig. 9), but not in patient 2, who had a somatic pathogenic variant only. A schematic representation of both atrophic and involutional changes is provided in Fig. 10.

Notably, in all four our patients, the indication to perform genetic germline test was based on the histological examination of the thyroid. This underlines the key role pathologists play in identifying these patients [50].

In this regard, immunohistochemistry can be a practical tool for screening familial tumors; for example, loss of PTEN stain in thyroid tumors can help to identify *PTEN* hamartoma tumor syndrome patients [2, 51].

Nevertheless, unlike tumor suppressor genes, the double-hit model of *DICER1* does not completely abolish protein function, but rather induces altered activity due to hotspot somatic mutations in the RNase IIIb domain. Moreover, the monoclonal anti-DICER1 antibody we used recognizes the amino acid sequence between proline 869 and histidine 1062. Since hotspot mutations occur in five precise sites, namely, E1705, D1709, G1809, D1810, and E1813, that antibody does not distinguish between the wild-type protein and that with hotspot mutations.

Studies conducted so far on both *DICER1*-mutated and wild-type tumors regarding DICER1 mRNA and protein expression levels have provided conflicting results. Paulsson et al. reported a downregulation of DICER1 mRNA and protein levels in *DICER1*-wild type thyroid tumors compared to normal thyroid parenchyma, especially in older patients and in oncocytic tumors [52]. Other authors confirmed that wild-type *DICER1* tumors have lower DICER1 mRNA levels, but they also demonstrated higher protein levels by Western blot and immunohistochemistry, especially in malignant and more aggressive tumors [53]. Conversely, in *DICER1*-driven tumors, DICER1 mRNA levels were reported to be increased, with higher levels in carcinomas than in adenomas [43]. Our immunohistochemical findings of increased DICER1 expression in mutated tumors (*p* < 0.001) appear to be in line with and support this pathogenic model. However, further studies are required to better define the role of DICER1 stain and its potential diagnostic use in identifying tumors with *DICER1* alterations. Without antibodies that recognize hotspot mutations of the IIIb domain, the stain is expected to be positive both in the presence of normal and mutated protein. For this reason, the ability to detect tumors harboring *DICER1* hotspot variants seems to rely on a different intensity of protein expression. This type of evaluation implies an inherent subjectivity issue and it is exposed to pre-analytical biases.

Although expressed in several tumor types, PRAME was found to be the most upregulated gene in pituitary blastoma, a tumor that is part of the *DICER1*-associated tumor spectrum [54]. A subsequent immunohistochemical study on various *DICER1*-driven lesions highlighted how PRAME was generally poorly expressed or negative in multinodular goiter nodules or in well-differentiated thyroid tumors [55]. In the context of *DICER1*-associated tumors, PRAME positivity was more expression of malignant progression rather than *DICER1* alterations [55]. Our results are in line with these conclusions as the expression was limited to tumors IA and IVB, which showed the most extensive vascular invasion plus highest mitotic count and full high-grade morphology respectively.

In conventional pediatric thyroid tumors, Ki-67 index does not appear to impact on prognosis [56]. High proliferation rates are common in *DICER1*-associated tumors [25, 47], but for encapsulated non-invasive tumors, excellent outcome has been reported so far [47, 57, 58]. In a rare study comparing the size of *DICER1*-mutated tumors with a control group of similar age, the mean diameter of the former was found 1 cm larger than of the latter (*p* = 0.035) [59]. Similarly, *DICER1*-mutant thyroid nodules showed greater ultrasound diameter and volume growth than wild-type nodules [60] and transcriptional profiling of mutant tumors showed upregulation of genes implicated in cell proliferation compared to non-neoplastic and hyperplastic thyroid lesions [43]. In Chernock’s series, patients who developed metastases and died of disease had *DICER1*-associated PDTC with an exceptionally high number of mitosis: 40 and 37/10 high power field as per original data [13]. In Whaley’s series, the only patient who died of disease and who had lymph node and lung metastases at presentation had 37 mitoses/2 mm^2^ as per original data. For this reason, in pediatric tumors or more specifically in *DICER1*-associated tumors, a higher mitotic cut-off should be considered to identify patients with a worse prognosis [9] and specific criteria for defining high-grade tumors in the pediatric population are required.

A greater number of patients with longer follow-up time will help to identify the most aggressive *DICER1*-driven tumors and to determine the most appropriate treatment for these patients.

## Conclusions

The current study has several limitations that deserve a thorough discussion. First, we reported a limited number of cases with a relatively short follow-up time, considering that late relapses are a possibility. Patient 2 had a lymph node recurrence 8 months after surgery that was confirmed histologically at another hospital. However, it was not possible to review the slides nor to perform molecular analysis. Finally, due to technical issues (decalcifying procedure), the molecular analysis of tumor IVB was unsuccessful.

Nevertheless, a series of preliminary data were provided even though further validation is obviously required.

The morphological spectrum of *DICER1*-associated tumors is broader than originally thought. Tumor architecture can be macrofollicular, microfollicular, or mixed, with papillary and cystic components. The presence of intratumoral nodules with STI growth pattern and increased proliferative activity (tumor-in-tumor pattern) could be another feature of these tumors.

Pathologists play a crucial role in promoting biomolecular analyses aimed at identifying somatic and/or germline *DICER1* alterations. Atrophic and involutional changes deserve attention in this regard. However, the sensitivity and specificity of the various findings reported so far have not yet been sufficiently validated in independent studies.

Immunohistochemical stain for DICER1 protein seems to confirm the results of previous studies as higher protein levels have been reported in tumors with *DICER1* alterations. However, it is not yet clear whether, in addition to providing support for a pathogenetic model, immunohistochemistry could play a supportive role in detecting mutated tumors. This is the first study to explore that possibility and further validation is needed.

The *ATM*-*CHEK2*-*TP53* pathway appears to be frequently implicated in malignant progression of *DICER1*-driven tumors. However, non-mutational mechanisms (altered expression of the *DICER1* gene harboring the pathogenic hotspot variant) could be involved as well.

*DICER1-*associated tumors seems to have a proliferative advantage, and the mitotic cut-off that should be employed for prediction of clinical aggressiveness has yet to be defined, but is probably higher than the adopted thresholds; specific criteria that take into account the biology of pediatric and specifically *DICER1*-driven tumors are required.

## Supplementary Information

Below is the link to the electronic supplementary material.

Figure S1. DICER1-immunohistochemistry in the control cohort. Intense reactivity (3+) in a medullary carcinoma (A, B). Weak reactivity (1+) in diffuse hyperplasia (C). Intense positivity (3+) in the germinal centers of lymphoid follicles of lymphocytic thyroiditis (D). Moderate reactivity (2+) in a papillary carcinoma and a follicular adenoma, respectively (E, F) (PDF 2.74 MB)

Figure S2. Immunohistochemistry for p53, Ki-67 and PRAME. In the high-grade area of tumor IVA, p53 positivity showed a weak and variable pattern, either in intensity and distribution, which was interpreted as wild-type (A). In the same patient, tumor IVB showed instead intense and diffuse reactivity for p53 (B). Ki-67 proliferative index in the high-grade component of tumor IVA was 13% (C). Thyroglobulin showed a dot-like perinuclear and to a lesser extent microfollicular pattern in the high-grade area of tumor II (D). PRAME staining was negative in the high-grade component of tumor IVA (E) while in tumor IVB showed reactivity in approximately 20% of the nuclei (F) (PDF 15.1 MB)

Supplementary file3 (DOCX 23.5 KB)

## Data Availability

No datasets were generated or analysed during the current study.

## Declarations

As per Italian law (resolution March 1, 2012, Gazzetta Ufficiale n.72 of March 26, 2012), due to the retrospective nature of the study, utilization of anonymous data, and its lack of impact on patient management, Ethical Committee approval was waived. The study procedures adhered to ethical standards set by the institutional and national research committee, aligning with the principles outlined in the 1964 Helsinki declaration and subsequent amendments or equivalent ethical norms. This study was performed in accordance with the Strengthening the Reporting of Observational Studies in Epidemiology (STROBE) guidelines.
