# Supplementary figures and images for: Focal High-Grade Areas with a Tumor-in-Tumor Pattern: Another Feature of Pediatric DICER1-Associated Thyroid Carcinoma?
Source: Endocr Pathol. 2025 May 31;36(1):20. doi: 10.1007/s12022-025-09863-2 (PMC12126348; doi:10.1007/s12022-025-09863-2)

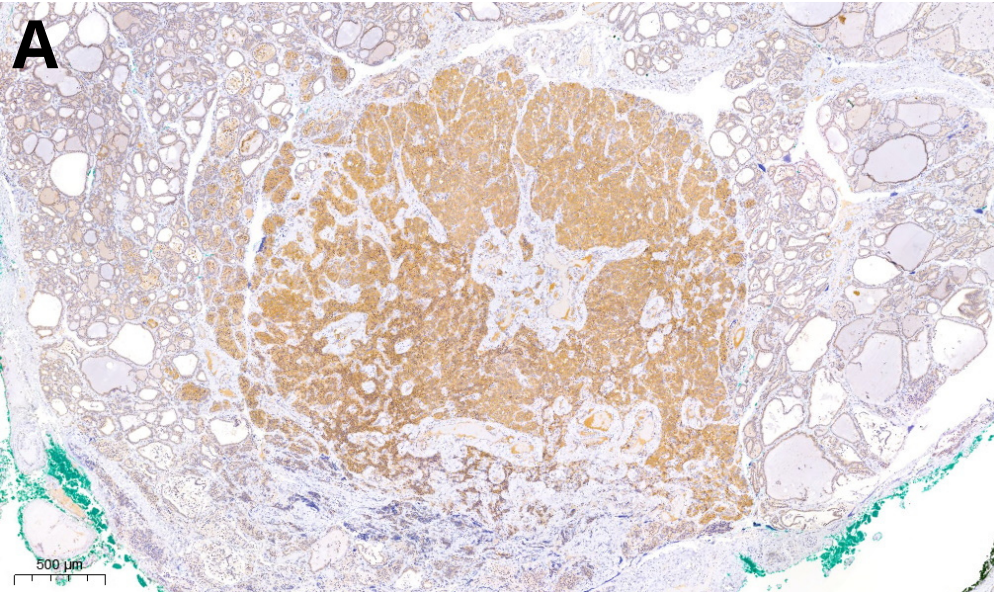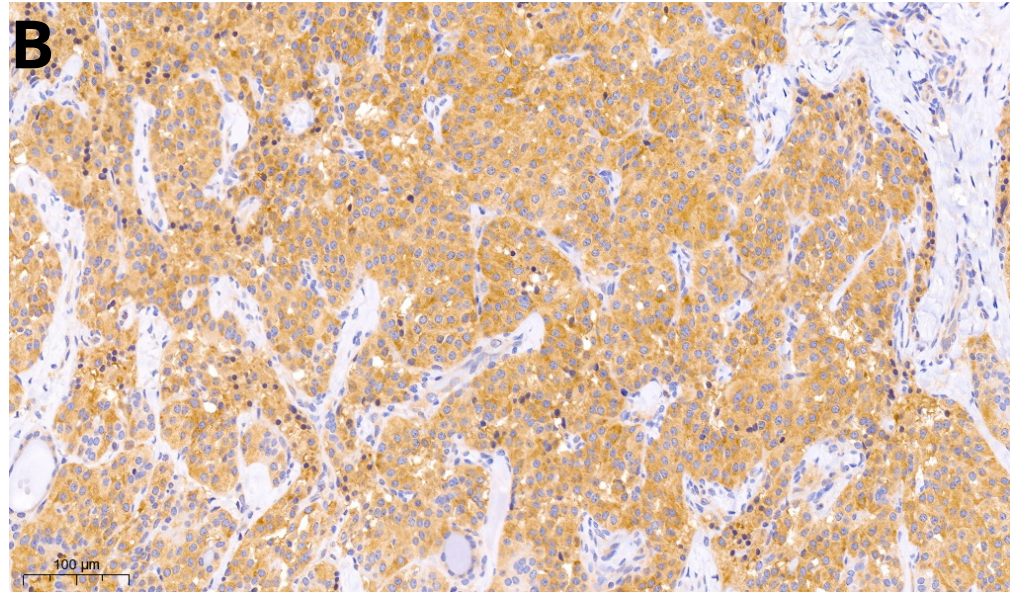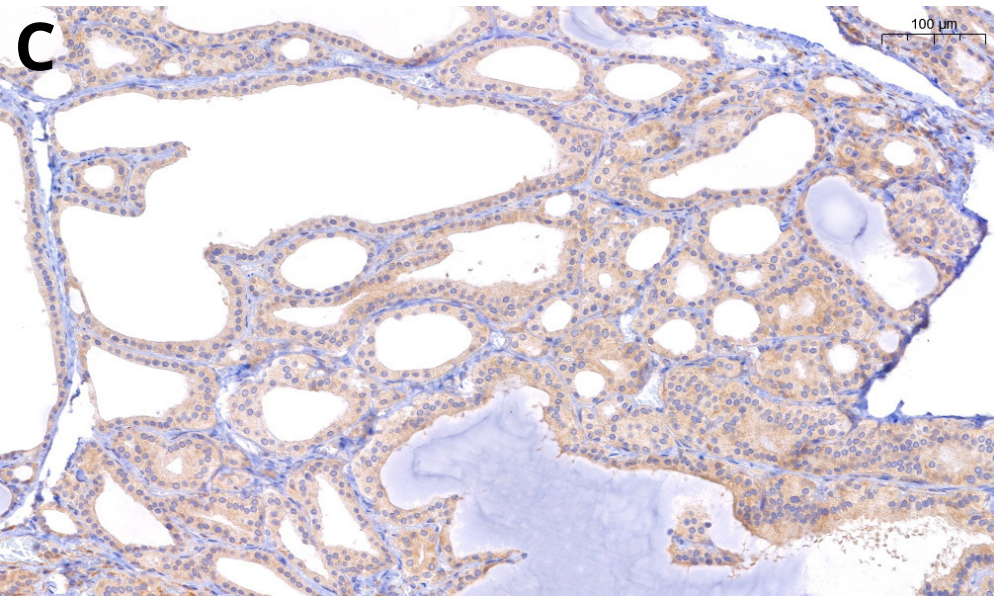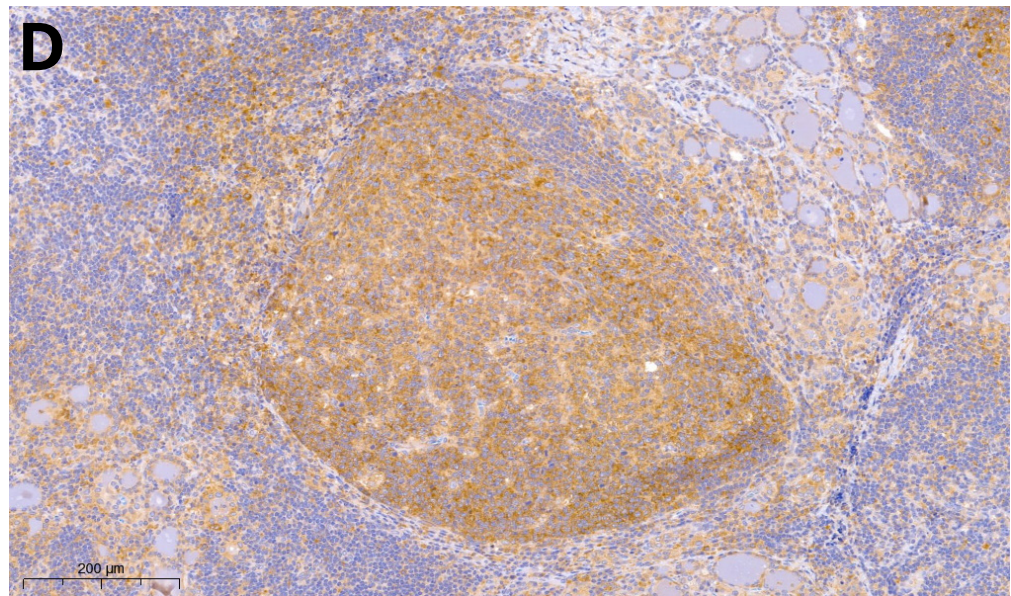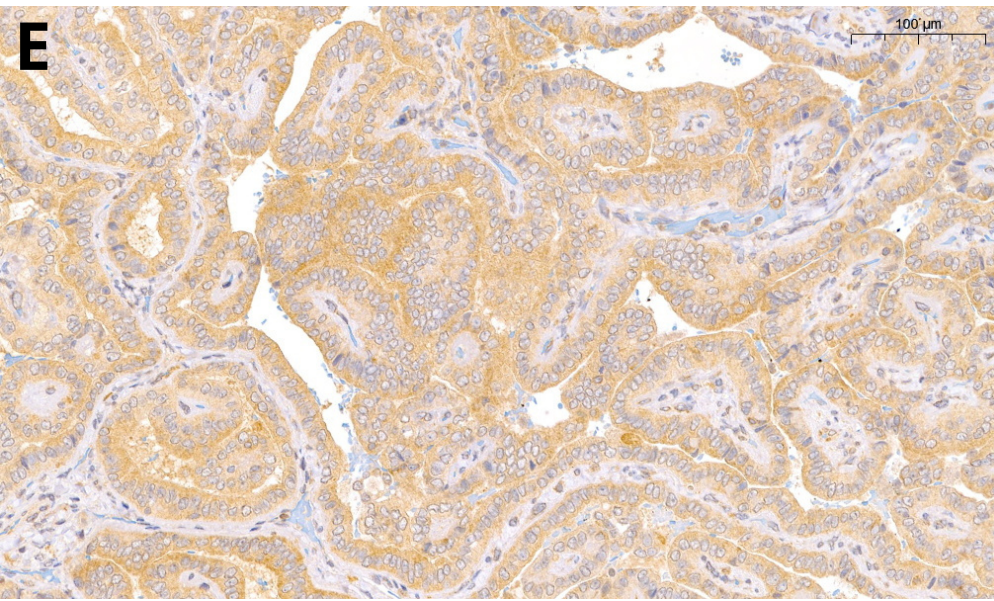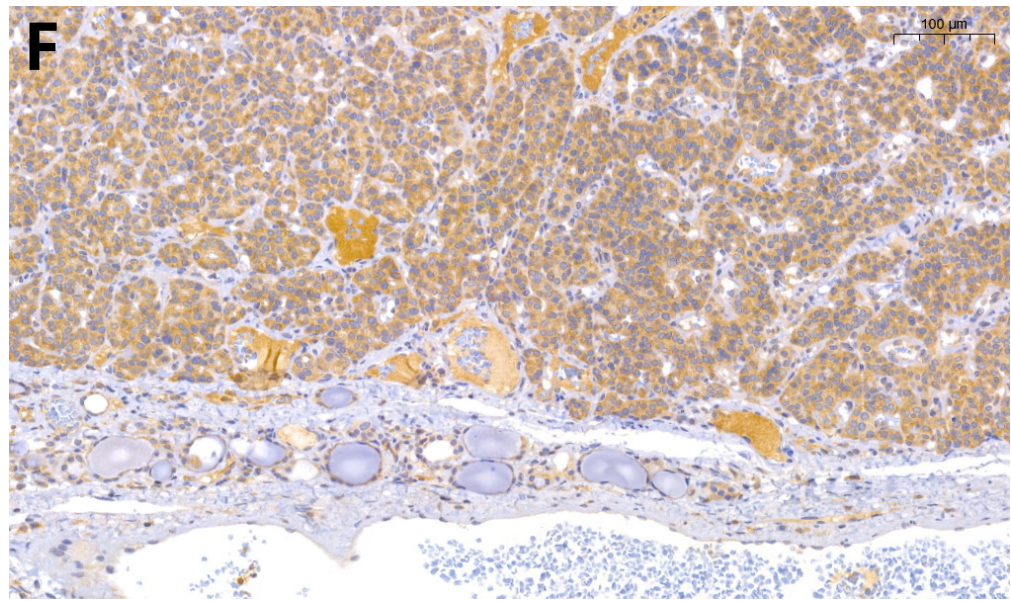

Supplement: Supplementary file 1 — Figure S1. DICER1-immunohistochemistry in the control cohort. Intense reactivity (3+) in a medullary carcinoma (A, B). Weak reactivity (1+) in diffuse hyperplasia (C). Intense positivity (3+) in the germinal centers of lymphoid follicles of lymphocytic thyroiditis (D). Moderate reactivity (2+) in a papillary carcinoma and a follicular adenoma, respectively (E, F) (PDF 2.74 MB) [file 12022_2025_9863_MOESM1_ESM.pdf]

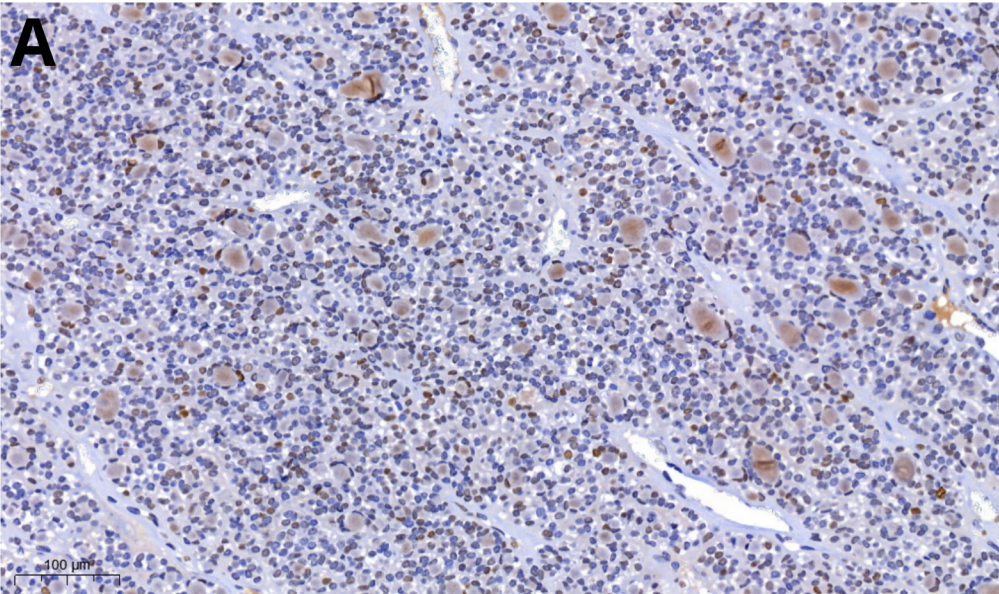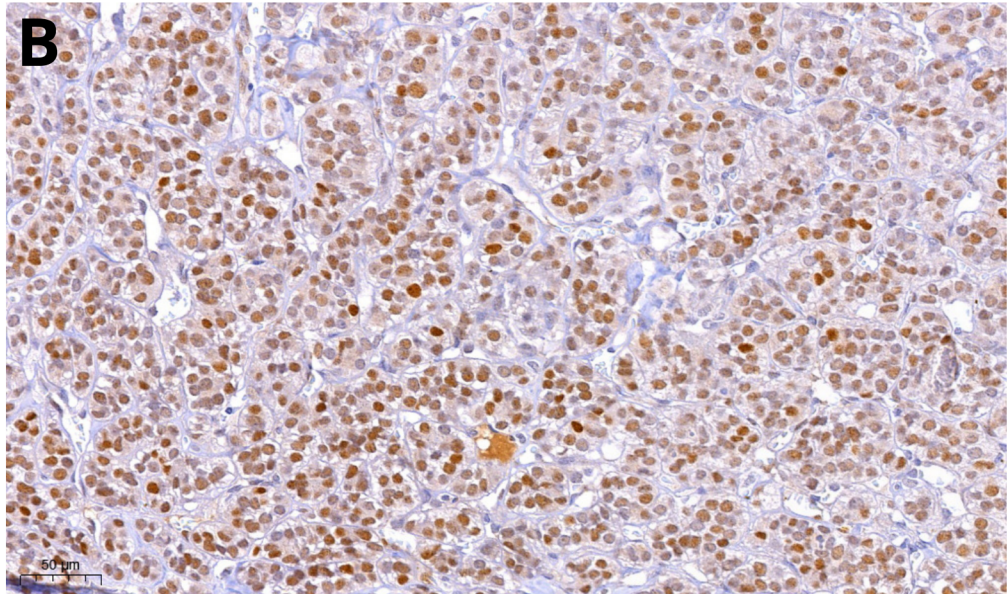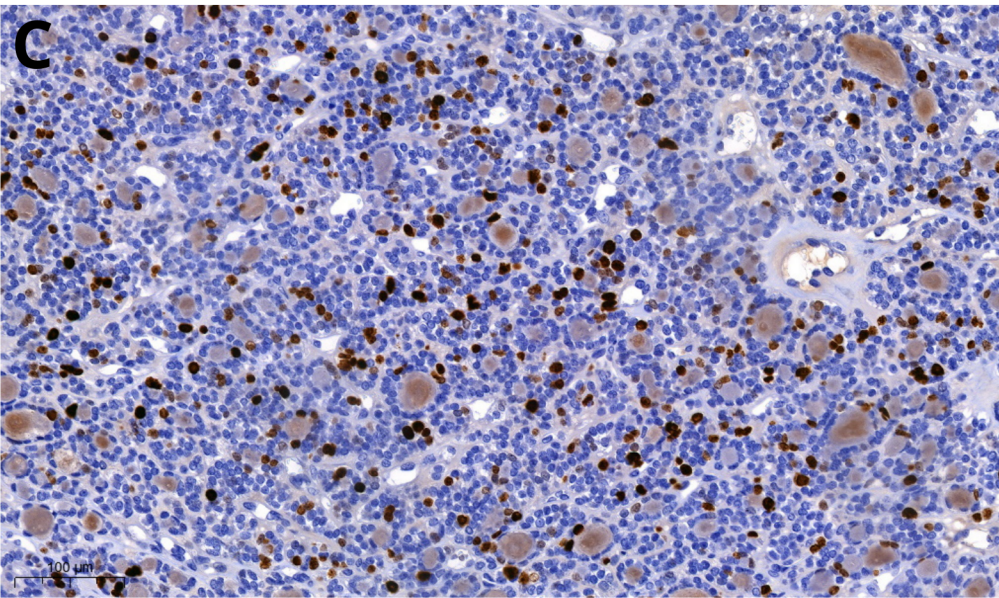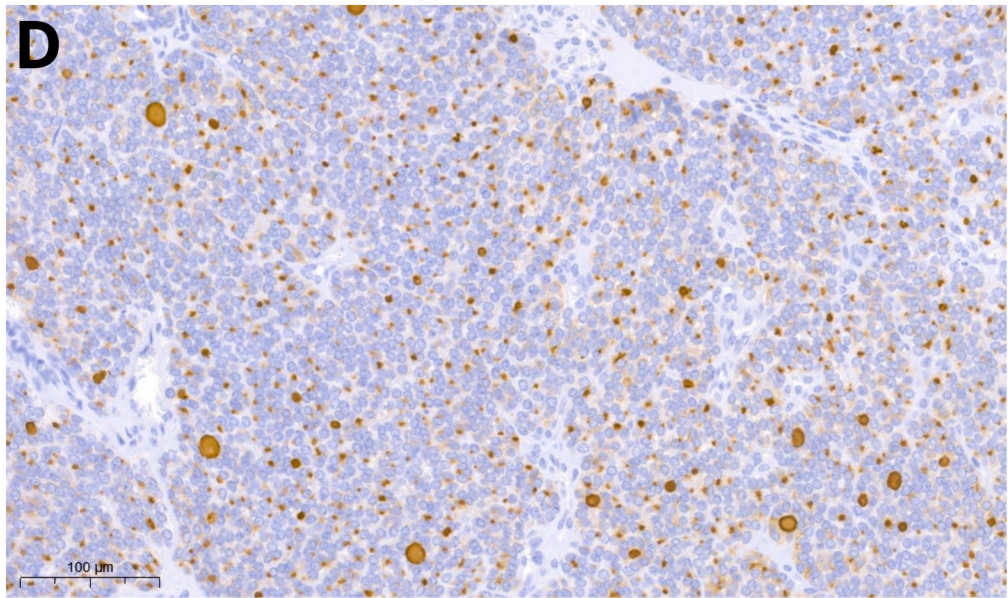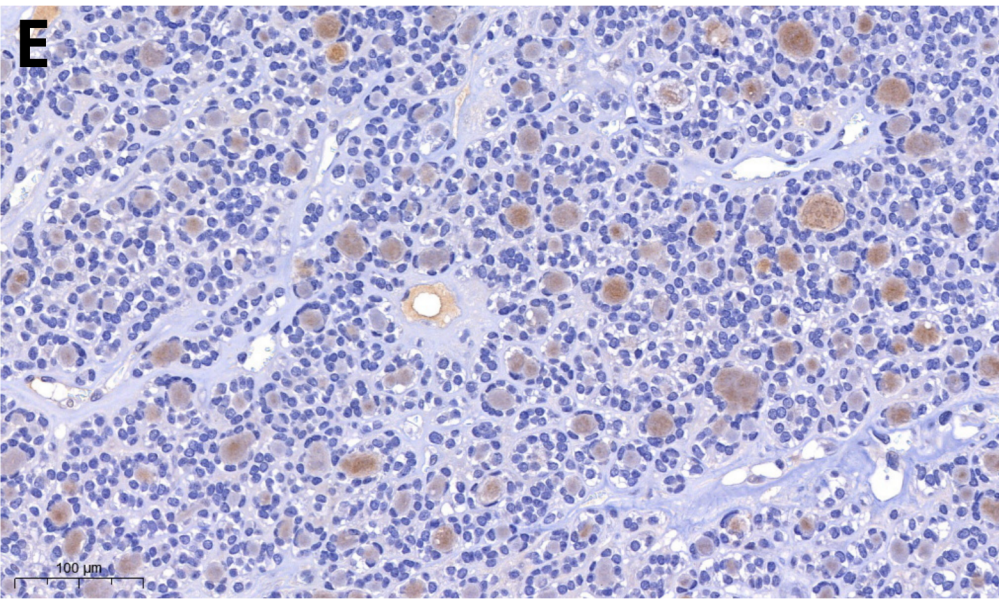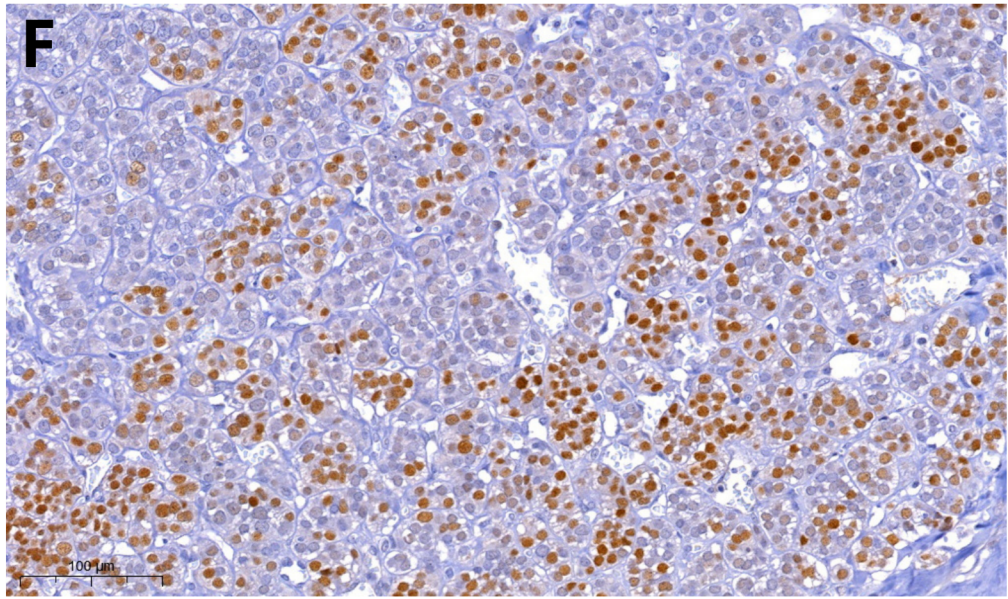

Supplement: Supplementary file 2 — Figure S2. Immunohistochemistry for p53, Ki-67 and PRAME. In the high-grade area of tumor IVA, p53 positivity showed a weak and variable pattern, either in intensity and distribution, which was interpreted as wild-type (A). In the same patient, tumor IVB showed instead intense and diffuse reactivity for p53 (B). Ki-67 proliferative index in the high-grade component of tumor IVA was 13% (C). Thyroglobulin showed a dot-like perinuclear and to a lesser extent microfollicular pattern in the high-grade area of tumor II (D). PRAME staining was negative in the high-grade component of tumor IVA (E) while in tumor IVB showed reactivity in approximately 20% of the nuclei (F) (PDF 15.1 MB) [file 12022_2025_9863_MOESM2_ESM.pdf]
