# Supplementary material for: Focal High-Grade Areas with a Tumor-in-Tumor Pattern: Another Feature of Pediatric DICER1-Associated Thyroid Carcinoma?
Source: Endocr Pathol. 2025 May 31;36(1):20. doi: 10.1007/s12022-025-09863-2 (PMC12126348; doi:10.1007/s12022-025-09863-2)
Supplement: Supplementary file 3 — Supplementary file3 (DOCX 23.5 KB) [file 12022_2025_9863_MOESM3_ESM.docx]

| **Supplementary Table 1**. List of genes included in the THYROSAN panel | |
| --- | --- |
| *Gene* | *National Center for Biotechnology Information (NCBI) reference sequence.* |
| *AKT1* | NM_001014431 |
| *AKT3* | NM_005465 |
| *ALK* | NM_004304 |
| *APC* | NM_000038 |
| *ARID1B* | NM_001374828 |
| *ATM* | NM_000051 |
| *ATR* | NM_001184 |
| *AXIN1* | NM_003502 |
| *AXIN2* | NM_004655 |
| *BRAF* | NM_004333 |
| *BRCA1* | NM_007294 |
| *BRCA2* | NM_000059 |
| *CDH1* | NM_004360 |
| *CDKN1B* | NM_004064 |
| *CDKN2A* | NM_000077 |
| *CHEK2* | NM_007194 |
| *CRTC1* | NM_015321 |
| *CTNNB1* | NM_001904 |
| *DAXX* | NM_001141970 |
| *DICER1* | NM_177438 |
| *DUOX2* | NM_001363711 |
| *EGFR* | NM_005228 |
| *EIF1AX* | NM_001412 |
| *ERBB2* | NM_004448 |
| *ERBB3* | NM_001982 |
| *ERBB4* | NM_005235 |
| *ESR2* | NM_001437 |
| *EWSR1* | NM_005243 |
| *EZH1* | NM_001991 |
| *FGFR1* | NM_023110 |
| *FGFR2* | NM_000141 |
| *FGFR3* | NM_000142 |
| *GNAS* | NM_000516 |
| *GREM1* | NM_013372 |
| *HABP2* | NM_004132 |
| *IDH1* | NM_005896 |
| *IDH2* | NM_002168 |
| *JAK1* | NM_002227 |
| *JAK2* | NM_004972 |
| *KIT* | NM_000222 |
| *KLLN* | NM_0011226049 |
| *KMT2A* | NM_001197104 |
| *KMT2D* | NM_003482 |
| *KRAS* | NM_004985 |
| *LTK* | NM_002344 |
| *MAML2* | NM_032427 |
| *MAP2K1* | NM_002755 |
| *MAP2K2* | NM_030662 |
| *MED12* | NM_005120 |
| *MEN1* | NM_130799 |
| *MET* | NM_000245 |
| *MSH2* | NM_000251 |
| *MTOR* | NM_004958 |
| *MUTYH* | NM_001048174 |
| *MYC* | NM_002467 |
| *NDUFA13* | NM_015965 |
| *NF1* | NM_001042492 |
| *NF2* | NM_000268 |
| *NRAS* | NM_002524 |
| *NRG1* | NM_013964 |
| *NTRK1* | NM_002529 |
| *NTRK2* | NM_006180 |
| *NTRK3* | NM_001012338 |
| *PARG* | NM_003631 |
| *PAX8* | NM_003466 |
| *PDGFRA* | NM_006206 |
| *PIK3CA* | NM_006218 |
| *POLD1* | NM_002691 |
| *POLE* | NM_006231 |
| *POT1* | NM_015450 |
| *PPM1D* | NM_003620 |
| *PRKAR1A* | NM_212471 |
| *PTEN* | NM_000314 |
| *PTK2* | NM_001352702 |
| *RB1* | NM_000321 |
| *RBM10* | NM_001204468 |
| *RET* | NM_020975 |
| *ROS1* | NM_002944 |
| *SDHA* | NM_004168 |
| *SDHB* | NM_003000 |
| *SDHC* | NM_003001 |
| *SDHD* | NM_003002 |
| *SEC23B* | NM_006363 |
| *SLC34A2* | NM_006424 |
| *SLC5A5* | NM_000453 |
| *SMAD4* | NM_005359 |
| *SOX2* | NM_003106 |
| *SPOP* | NM_001007228 |
| *SRGAP1* | NM_020762 |
| *SRRM2* | NM_016333 |
| *STK11* | NM_000455 |
| *TERT* | NM_198253 |
| *TG* | NM_003235 |
| *THADA* | NM_022065 |
| *TP53* | NM_000546 |
| *TPO* | NM_001206744 |
| *TSHR* | NM_000369 |
| *TTF1* | NM_007344 |
| *TTF2* | NM_003594 |
| *TTN* | NM_001267550 |
| *USF3* | NM_001009899 |
| *WRN* | NM_000553 |
